# Structural and functional mapping of Rtg2p determinants involved in retrograde signaling and aging of *Saccharomyces cerevisiae*

**DOI:** 10.1371/journal.pone.0177090

**Published:** 2017-05-04

**Authors:** Rafaela Maria Rios-Anjos, Vittoria de Lima Camandona, Lucas Bleicher, Jose Ribamar Ferreira-Junior

**Affiliations:** 1 Escola de Artes, Ciências e Humanidades, Universidade de São Paulo, São Paulo, Brazil; 2 Departamento de Bioquímica e Imunologia, Instituto de Ciências Biológicas, Universidade Federal de Minas Gerais, Belo Horizonte, Minas Gerais, Brazil; Tulane University Health Sciences Center, UNITED STATES

## Abstract

In *Saccharomyces cerevisiae* mitochondrial dysfunction induces retrograde signaling, a pathway of communication from mitochondria to the nucleus that promotes a metabolic remodeling to ensure sufficient biosynthetic precursors for replication. Rtg2p is a positive modulator of this pathway that is also required for cellular longevity. This protein belongs to the ASKHA superfamily, and contains a putative N-terminal ATP-binding domain, but there is no detailed structural and functional map of the residues in this domain that accounts for their contribution to retrograde signaling and aging. Here we use Decomposition of Residue Correlation Networks and site-directed mutagenesis to identify Rtg2p structural determinants of retrograde signaling and longevity. We found that most of the residues involved in retrograde signaling surround the ATP-binding loops, and that Rtg2p N-terminus is divided in three regions whose mutants have different aging phenotypes. We also identified E137, D158 and S163 as possible residues involved in stabilization of ATP at the active site. The mutants shown here may be used to map other Rtg2p activities that crosstalk to other pathways of the cell related to genomic stability and aging.

## Introduction

In cells of the baker´s yeast *Saccharomyces cerevisiae* mitochondrial dysfunction triggers the transcription of many nuclear genes [1]. This transcriptional activation is modulated by a pathway of communication from mitochondria to the nucleus termed retrograde signaling [2, 3]. Because *S*. *cerevisiae* can grow devoid of mitochondrial DNA (mtDNA), this pathway was extensively explored in this organism. A genome-wide approach in cells lacking mtDNA revealed that when retrograde signaling is activated cells undergo a metabolic remodeling by increasing peroxisomal and mitochondrial activities required to glutamate synthesis [4]. In wild type cells, when glutamate reach sufficient levels retrograde signaling transcriptional response is down-regulated [5]. As glutamate is necessary for the synthesis of several amino acids that include glutamine, which in turn is required for the synthesis of purines and pyrimidines, the communication from mitochondria to the nucleus ensures cell´s metabolism will provide enough biosynthetic precursors to guarantee its replication [3].

At the molecular level retrograde signaling was characterized by analyzing the transcriptional activation of the gene *CIT2*, that encodes the peroxisomal isoform of citrate synthase [6]. When mitochondrial activity and glutamate levels are low, a heterodimeric transcription factor complex composed of the proteins Rtg1p and Rtg3p translocate to the nucleus to activate the transcription of *CIT2* [7]. Rtg1p and Rtg3p belong to the class of bHLH/Zip (basic helix-loop-helix leucine zipper) transcription factors and bind to the element GTCAC (UASr) present in *CIT2* promoter. The migration of this transcriptional complex requires the protein Rtg2p that associates to Mks1p, the negative modulator of the pathway [8, 9]. When mitochondrial activity and glutamate levels are high, Mks1p is released from Rtg2p, phosphorylated by an unknown kinase, and binds to 14-3-3 proteins Bmh1p/Bmh2p to execute its repression function on the pathway [9]. This keeps Rtg3p also phosphorylated, sequestered in the cytoplasm with Rtg1p, and *CIT2* transcription at basal level [8].

The proteins Rtg2p and Mks1p form the minimal binary switch for the regulation retrograde signaling [10]. However, this pathway also crosstalks with other pathways of the cell such as the target of rapamycin (TOR) pathway [11], the amino acid SPS (Ssy1-Ptr3-Ssy5) sensor system [12], and Ras signaling [13]. In particular, Rtg2p is a protein involved in several cellular activities that extend this crosstalk to the modulation of genomic stability and replicative aging.

Rtg2p is a protein involved in the expansion of trinucleotide repeats (TNR) in the nucleus, and also is present in the chromatin remodeling complex SLIK, and modulates the production of circles of rDNA. TNR instability is a tract involved in etiology of several diseases such as fragile X syndrome and Huntington’s disease. In *S*.*cerevisiae rtg2*Δ cells the expansion of CTG•CAG repeats showed a modest increase in rate, indicating a role for Rtg2p in genomic stability [14]. Besides this modulation of TNR expansion Rtg2p was also found in the nucleus bound to the SAGA (Spt-Ada-Gcn5) histone acetyltransferase complex [15]. The Rtg2p-SAGA complex was named SLIK (SAGA-like), and it connects retrograde signaling to chromatin remodeling. Another important activity of Rtg2p is that it suppresses the production of extrachromosomal ribosomal-DNA (rDNA) circles that accumulate in yeast cells as they age [16]. This accumulation is also a form of genomic instability that reduces longevity and can even kill cells when in very high levels [17]. Indeed, *rtg2*Δ strain have lower replicative lifespan when compared to wild type [13]. The function of Rtg2p in the suppression of rDNA circles is observed only when the protein is not involved in propagating retrograde signaling [16], as yeast cells lacking mitochondrial DNA (petite ρ^0^) accumulate high levels of extrachromosomal rDNA episomes [18].

When retrograde signaling is transduced from mitochondria to the nucleus, it requires the putative ATP-binding domain located at the N-terminal of Rtg2p [9]. This domain is predicted to belong to the ASKHA (Acetate and Sugar Kinases, Hsp70, and Actin) protein superfamily [19], that includes acetate kinases, eukaryotic hexokinases, glycerol kinase and exopolyphosphatases, the heat shock protein hsp70, and actin. Multiple sequence alignment of this superfamily located in this domain five conserved regions termed PHOSPHATE 1, PHOSPHATE 2, CONNECT 1, CONNECT 2 and ADENINE that are characteristic of the ATP-binding fold, where the nucleotide makes favorable interactions with Mg^2+^ ion coordinated by conserved aspartate residues [20]. This common fold is used for inorganic polyphosphate hydrolysis in exopolyphosphatases and transfer of phosphoryl group from ATP in sugar and acetate kinases. Although mutations in this domain impair Rtg2p function in retrograde response there is no detailed structural and functional map of this protein that correlates its N-terminal domain with longevity. Here we use amino acid correlation analysis and site-directed mutagenesis to identify structural determinants in Rtg2p involved in retrograde signaling and in aging of *S*. *cerevisiae*. Our results demonstrate that the N-terminal domain is very important to the function of Rtg2p, both in longevity and retrograde response, locates amino acid residues that may be involved in coordination of Mg^2+^ ion and stabilization of ATP in the active site, and also show there are structural determinants in Rtg2p that control longevity in both dependent or independent manner of the communication from mitochondria to the nucleus.

## Materials and methods

### Yeast strains, culture media and growth conditions

All strains used in this study were derivatives of PSY142 (ρ^+^ *MAT*α *leu2 lys2 ura3*::*CIT2-LacZ*). This strain has integrated the reporter *CIT2-lacZ* into the genome [9]. Cells were grown at 30°C in YPD medium (1% yeast extract, 2% peptone, and 2% dextrose), YPR (1% yeast extract, 2% peptone, and 2% raffinose), YPEG (1% yeast extract, 2% peptone, 2% ethanol and 2% glycerol), and YNBD (0.17% yeast nitrogen base, 0.5% (NH_4_)_2_SO_4_, 2% dextrose) with or without glutamate (final concentration 0.02%). Yeast cells were transformed as described previously [21]. To generate ρ^0^ strains, ρ^+^ cells were cultured for about 40 generations in liquid YPD medium supplemented with 25 μg/mL of ethidium bromide.

### Construction of *rtg2*Δ strain

The strain carrying *rtg2*Δ mutation was constructed by one step gene disruption using T-urf13-KanMX cassette. To construct this cassette, T-urf13 gene was removed from YIpTW [22] and the 2.6 kb fragment cloned into *Hin*dIII site of YIplac128 [23] generating YIplac128-T-urf13 vector. The gene KanMX from the plasmid pYM-N18 [24] was removed with enzymes *Bam*HI e *Sac*I, and the 1.5 kb fragment cloned into YIplac128-T-urf13 to produce YIplac128-T-urf13-KanMX. To disrupt *RTG2*, the cassette T-urf13-KanMX was amplified by PCR with the oligonucleotides rtg2-T-urf13-KanMX-F and rtg2-T-urf13-KanMX-R (S1 Table), and the product transformed into yeast. Cells were selected o YPD containing geneticin (G418, 200 μg/mL). To confirm the integration within *RTG2* locus genomic DNA was extracted from transformants and PCR reactions were performed with the oligonucleotides pairs rtg2-597up-F/KanB and KanC/rtg2-2217down-F.

### Construction of *RTG2* mutants by site-directed mutagenesis

The mutants alleles were obtained by site-directed mutagenesis performed by overlap extension PCR [25]. Briefly, for every mutant produced, two independent PCR reactions were performed with oligonucleotides pairs RTG2-F/mutagenic primer-R and mutagenic primer-F/RTG2-R (S1 Table). The products were submitted to gel electrophoresis, purified and combined in a PCR reaction without primers for 10 cycles. Finally, oligonucleotides RTG2-F and RTG2-R were added to proceed the reaction for 30 more cycles to generate the full length product with the desired mutation. PCR products were digested with *Bam*HI and *Hind*III, and cloned into pGEM-3Zf(+). DNA sequencing was conducted to confirm the identity of the alleles.

### Production of *RTG2* mutants by gene replacement

The mutants cloned in pGEM3zf(+) were digested with *Bam*HI and *Hind*III, transformed into *rtg2*Δ cells in the presence of lithium acetate [21], and selected on YPD plates containing methomyl (final concentration 6 mM) [22]. The DNA of several transformants was extracted to confirm the integration locus by PCR using the primers rtg2-597up-F/G337-R (S1 Table).

### Auxotrophy assays and growth curves

Cells were grown until A_600_ = 1 and subjected to four serial dilutions (10^−1^, 10^−2^, 10^−3^, and 10^−4^) and an aliquot of 3μl of each dilution was deposited on YNBD plates with or without glutamate. The plates were incubated at 30°C for 3 days and photographed to observe the differential growth of the mutants. Growth curves were constructed by measuring the A_600_ every hour in a microplate reader INFINITY PRO 200 (Tecan) with constant agitation of 320 rpm at 30°C for 24h.

### β-galactosidase assays

Yeast cells were inoculated and grown in YNBD without glutamate until A_600_ 0.6–0.8. The preparation of cell extracts and β-galactosidase assays were conducted as described previously [26]. All protein quantifications were performed according to Bradford [27]. Assays were conducted in triplicate, and independent experiments were carried out 2–3 times.

### Yeast replicative lifespan

Replicative lifespan was determined as described previously [28]. Briefly, 50 virgin cells from an overnight culture were aligned on a YPD plate. After each cell division daugther cells were separated from their mothers. The number of daughter cells (generations) that each mother cell was able to bud until stop dividing was scored. During the day the cells were grown at 30°C, but incubated overnight at 4°C. Replicative lifespan assays were repeated at least twice and the Mann-Whitney test was used to measure the statistical significance.

### Residue conservation and correlation analysis

Rtg2p is part of the Ppx-GppA protein family in PFAM (PF02541) that belongs to the ASKHA superfamily [29]. An alignment of 5020 sequences from Rtg2p homologs was obtained from PFAM [30]. These sequences were aligned and filtered, sequences with less than 80% coverage and 20% identity when compared to Rtg2p were excluded. After this procedure all sequences were compared to each other to remove similar sequences, if two sequences of the alignment had more than 80% identity, the smallest was removed. The resulting final alignment contained 581 sequences. Residue specific correlations and conservation were calculated using PFstats as described previously [31].

### Molecular modeling

Molecular modeling of Rtg2p was performed in five independent servers, Robetta [32], SWISS-MODEL [33], Phyre2 [34], ESyPred3D [35] and I-TASSER [36] using default settings. The models obtained were validated by analysis of the Ramachandran diagram (S2 Table) and through Verify3D program (S3 Table) [37]. The best model showed 90.2% of residues in favorable regions in the Ramachandran diagram and more than 80% of coherence in Verify3D, that are indicators of good quality, and was obtained with Robetta server. Rtg2p model was refined with the 3Drefine for hydrogen bonding network optimization and atomic-level energy minimization [38]. The final model was deposited in the Protein Model Database (PMDB), accession code PM0080565. Figures were generated using PyMOL (https://www.pymol.org/).

## Results

### Rtg2p molecular model is a three domain structure similar to the crystal structure of a putative exopolyphosphatase of the ASKHA superfamily

**Fig 1:**
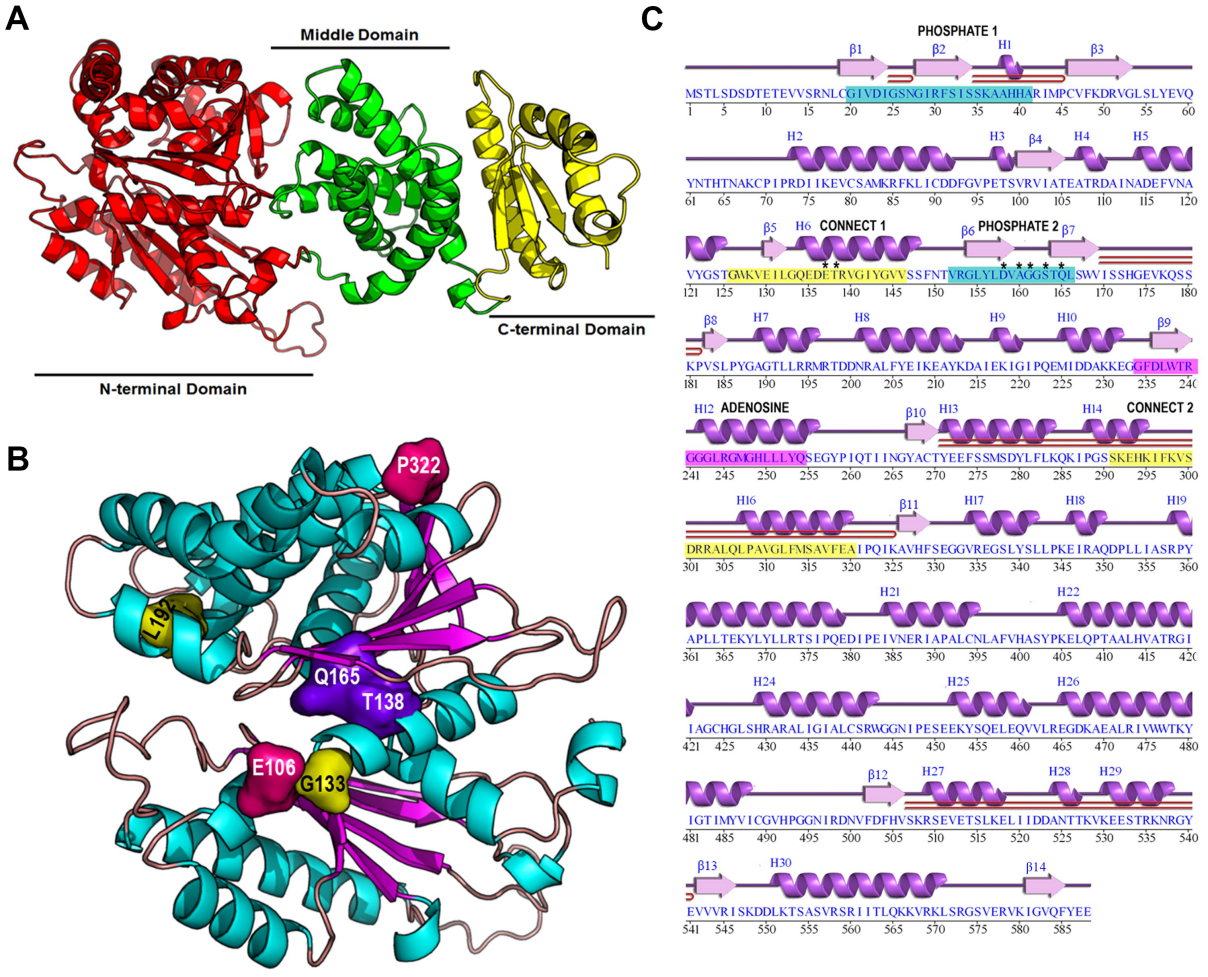
Domain organization of Rtg2p structural model, location of coevolved residues, and ATP-binding motifs. **(A)** Structure of the Rtg2p model obtained with the server Robetta [32]. Domain organization is indicated. **(B)** Location of residue communities obtained by DRCN on Rtg2p N-terminal Domain. Community 1 is represented in pink, community 2 in yellow, and community 3 in purple **(C)** Rtg2p ‘wiring diagram’ generated with PDBsum [41] highlighting the five sequence motifs involved in ATP binding (PHOSPHATE 1 and PHOSPHATE 2 (blue), CONNECT 1 and CONNECT 2 (yellow), and ADENOSINE(pink)). Residues on these motifs that were modified by site-directed mutagenesis are marked with an asterisk, and those making β-hairpins are represented by lines underneath.

There is no published crystal structure of Rtg2p given the difficulties found in its purification from bacteria due to low solubility and partitioning into inclusion bodies [39]. For this reason, a 3D structure model of Rtg2p was generated in the server Robetta (Fig 1A) that used the crystal structure of the putative exopolyphosphatase of *Agrobacterium fabrum* (strain C58 / ATCC 33970) (PDB ID 3HI0) as a domain reference structure. The superposition of 440 structurally equivalent C_α_ atoms of both structures with a root mean square error of 2.3Å, as revealed by TopMatch [40], indicates the quality of our structural model of Rtg2p (S1 Fig).

Rtg2p is a 588 amino acids long protein whose modeled structure is organized in three domains (Fig 1A). The N-terminal domain (amino acids 1–352, Fig 1A, red domain) contains two smaller domains each with a βββαβαβα fold that is characteristic of the ASKHA superfamily [29] of proteins that include exopolyphosphatases and phosphotransferases. This domain corresponds to the Pfam family PF02451 of Pfam database. The middle domain (amino acids 353–496, Fig 1A, green domain) is an all α structure with six α helices that compose a six-helix claw, and a structural comparison with TopSearch [42] identified the catalytic core of the human cAMP phosphodiesterase PDE4B2B as its structural homolog. Finally, the C-terminal domain (amino acids 497–588, Fig 1A, yellow domain) contains a small β-sheet whose side chain residues make hydrophobic contacts with residues from Helix 30 on one side, and polar interactions with charged residues of two small helices on the other side. There is no protein domain assigned for both Middle and C-terminal domains of Rtg2p in any Pfam family. Indeed, for amino acids residue correlation analysis only the N-terminal domain could provide the alignment used in this work.

### Decomposition of Residues Correlation Networks identified relevant functional residues in Rtg2p

The N-terminal domain of Rtg2p is important for its function. Mutations in this domain disrupt the transduction of mitochondrial signals of retrograde signaling [9]. In order to characterize this domain structurally we performed a bioinformatics analysis using the program PFstats. This analysis is termed Decomposition of Residues Correlation Networks (DRCN) [31], and it allows the identification of residues that coevolved in the protein. The input of PFstats is the alignment of the Pfam family PF02541, in which Rtg2p is a member, and the output is a set of communities of coevolved residues.

DRCN analysis of PF02541 family produced three communities of coevolved residues: E106-P322, L192-G133, and A138-E165 (Fig 1B). Rtg2p contains most of the residues that coevolved in the family except that T138 and Q165, which are in close contact, are different of those present in community 3 in equivalent positions, where the majority of the family members contain alanine and glutamate, respectively (Table 1).

**Table 1:** Conserved amino acid residues on equivalent positions of the ASKHA family members.

UNIPROT code	Conserved residues in equivalent positions	
**ASKHA family**^1^	**A263**	**E423**
RTG2_YEAST	T138	Q165
A9CJF9_AGRT5	A125	E152
O67040_AQUAE	G120	E148
PPX_ECO57	A122	E150
PPX_ECOLI	A122	E150
Q11YA9_CYTH3	A114	E143
Q8G5J2_BIFLO	A118	E149

^1^Conservation of the ASKHA family in the filtered alignment of DRCN analysis.

PFstats also produced a residue conservation analysis of the family. In this analysis only residues with conservation above 90% were considered (S4 Table). In particular, at the alignment position 418, in 99.8% of the sequences there is a glycine residue whereas Rtg2p contains alanine in an equivalent position, indicating a divergence of the family. It is also worth to mention that in Rtg2p primary structure the residues located at positions 204, 262, and 495 of the alignment, which are, respectively, threonine glutamate and glycine, are found in 100% of the sequences. In addition, some conserved residues and those of community 3 locate to two of the five sequence motifs involved in ATP binding, named PHOSPHATE 1, PHOSPHATE 2, CONNECT 1, CONNECT 2, and ADENOSINE [43]. The residues E137, and T138 (that coevolved with Q165 in community 3) are located in CONNECT 1 and D158, A160, G161, S163, and Q165 are located in PHOSPHATE 2 (Fig 1C). Taken together both coevolved residues (Fig 1B) and conserved residues (S2 Fig) located at the N-terminal domain at critical structural motifs are suitable candidates for site-directed mutagenesis to analyze the structural determinants of Rtg2p function.

### The mutation *RTG2-L56G* render yeast cells auxotrophic for glutamate, impairs retrograde signaling but extends replicative longevity

**Fig 2:**
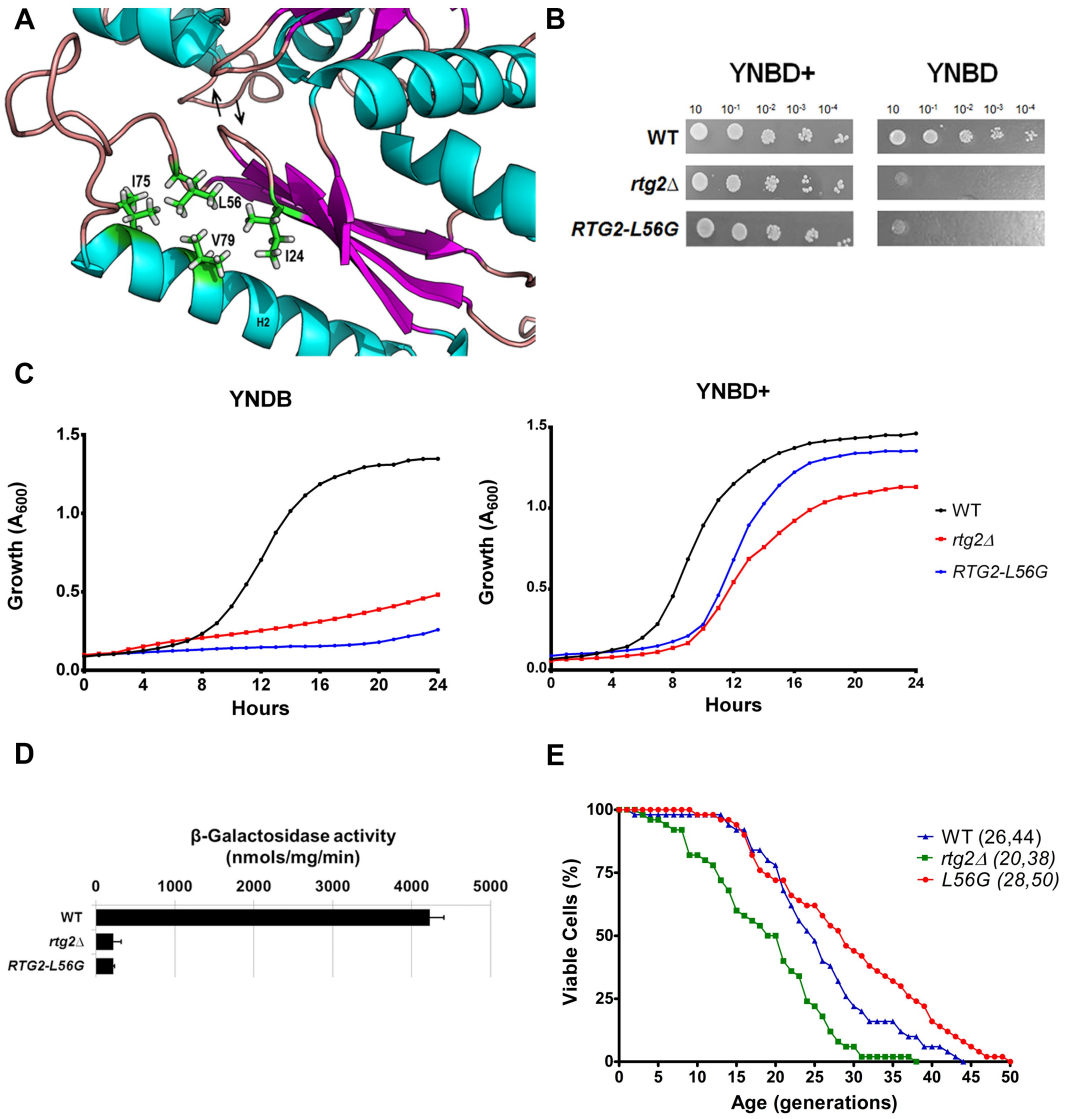
The mutation RTG2-L56G impair retrograde signaling, but extends replicative longevity. **(A)** Location of L56 residue on Rtg2p protein structural model. ATP-binding loops are indicated with arrows. **(B)** Mutation L56G in Rtg2p render glutamate auxotrophy to yeast cells. Wild type, *rtg2*Δ, and *RTG2-L56G* strains were grown on YPD to A_600_ = 1 and four serial dilutions (10^−1^, 10^−2^, 10^−3^, and 10^−4^) were spotted on solid minimal media YNBD and YNBD+ (YNBD plus glutamate at 0.02%). The plates were incubated at 30°C for three days when photographs were taken. **(C)** Growth performance of strains on minimal liquid medium. Wild type, *rtg2*Δ and *RTG2-L56G* strains were grown on YPD until saturation, and diluted to A_600_ = 0.1 in 200 μL of either YNBD or YBND+. The cells were incubated at 30°C, 160 rpm, for 24 h and growth monitored in a INFINITY PRO 200 (Tecan) microplate reader. **(D)** L56G mutation impairs retrograde signaling activation on minimal medium. Wild type, *rtg2*Δ and *RTG2-L56G* strains were grown on YNBD medium until A_600_ = 0.6 and cell extracts were prepared to analyze *CIT2-LacZ* expression. β-galactosidase assays were performed in triplicate as described in the Materials and Methods section and the activity normalized by total protein concentration. **(E)** Replicative lifespan is extended in the mutant *RTG2-L56G*. Fifty cells of each strain were aligned on YPD and daughter cells were removed from mothers to construct survival curves from at least two independent experiments. The mean and maximum longevity are indicated between parentheses (mean, maximum). Statistical significance between samples is summarized in Table 2.

One of Rtg2p conserved residues is L56, which lies in a hydrophobic region in the vicinity of I24, I75 and V79 (Fig 2A). We hypothesized that L56 hydrophobic interactions would be necessary for protein activity, and to test this we generated the *RTG2-L56G* mutant by site-directed mutagenesis using overlap extension PCR and gene replacement. Cells with *RTG2* gene disrupted (*rtg2*Δ) are glutamate auxotrophs [5]. If the mutant produced a similar auxotrophy phenotype it could be verified by growing cells in YNBD medium in the presence or absence of glutamate, and, indeed, *RTG2-L56G* mutant cells are glutamate auxotrophs, when compared to *rtg2*Δ and wild type parent (Fig 2B). In order to confirm the result of the spot assay, *RTG2-L56G* mutant cells were grown in liquid medium and the A_600_ measured for 24h. In the presence of glutamate *RTG2-L56G* mutant cells grew as well as the wild type, but a growth curve very similar to that of the *rtg2*Δ mutant was obtained when cells were grown in the absence of glutamate (Fig 2C), indicating a clear auxotrophy.

The strains used in this work contain *CIT2-LacZ* as a reporter integrated into the genome. *CIT2* is a prototypical target gene of retrograde signaling that is induced when cells are grown in minimal medium YNBD, in the absence of glutamate [5]. This induction is impaired in *rtg2*Δ background. In order to analyze the effect of Rtg2p mutations on the activation of retrograde signaling and *CIT2* transcription we measured the β-galactosidase activity of the reporter expressed in cells grown in YNBD. The mutant *RTG2-L56G* showed a clear phenotype of *rtg2*Δ cells, being unable to activate retrograde signaling as the parent wild type that showed a *CIT2* expression higher than 30 fold (Fig 2D).

Prior work has shown that yeast cells lacking mitochondrial DNA (petite ρ^0^) have extended replicative lifespan (RLS) in glucose rich medium (YPD), when compared to the wild type (grande ρ^+^). This lifespan extension is abrogated by deletion of *RTG2* [13]. This effect was strain-dependent, and displayed three possible phenotypes: extended longevity for YPK9 strain, shorter lifespan for SP1-1 and A364A strains, and no change for W303-1A. In order to determine which was the case of PSY142 we generated a ρ^0^ mutant and observed no difference between petite and grande lifespans (S3 Fig). Although we did not find a longevity extension dependent on *RTG2* in our PSY142 petite strain, we did not exclude the possibility that our *RTG2* mutants may interfere with yeast longevity. Surprisingly, when compared to the wild type grande strain, the mutant *RTG2-L56G* showed an increase in RLS (Fig 2E).

Taken together, these results suggest that Rtg2p L56 residue is important for protein function that is possibly accomplished by hydrophobic interactions of L56 with some of its neighbor residues (I24, I75 and V79). These interactions may determine the phenotypes of glutamate prototrophy and retrograde signaling activation observed in wild type cells. Unexpectedly, this single amino acid substitution had a positive effect by extending replicative longevity of the cells.

### Interaction between the pair of coevolved residues T138 and Q165 determine Rtg2p function

**Fig 3:**
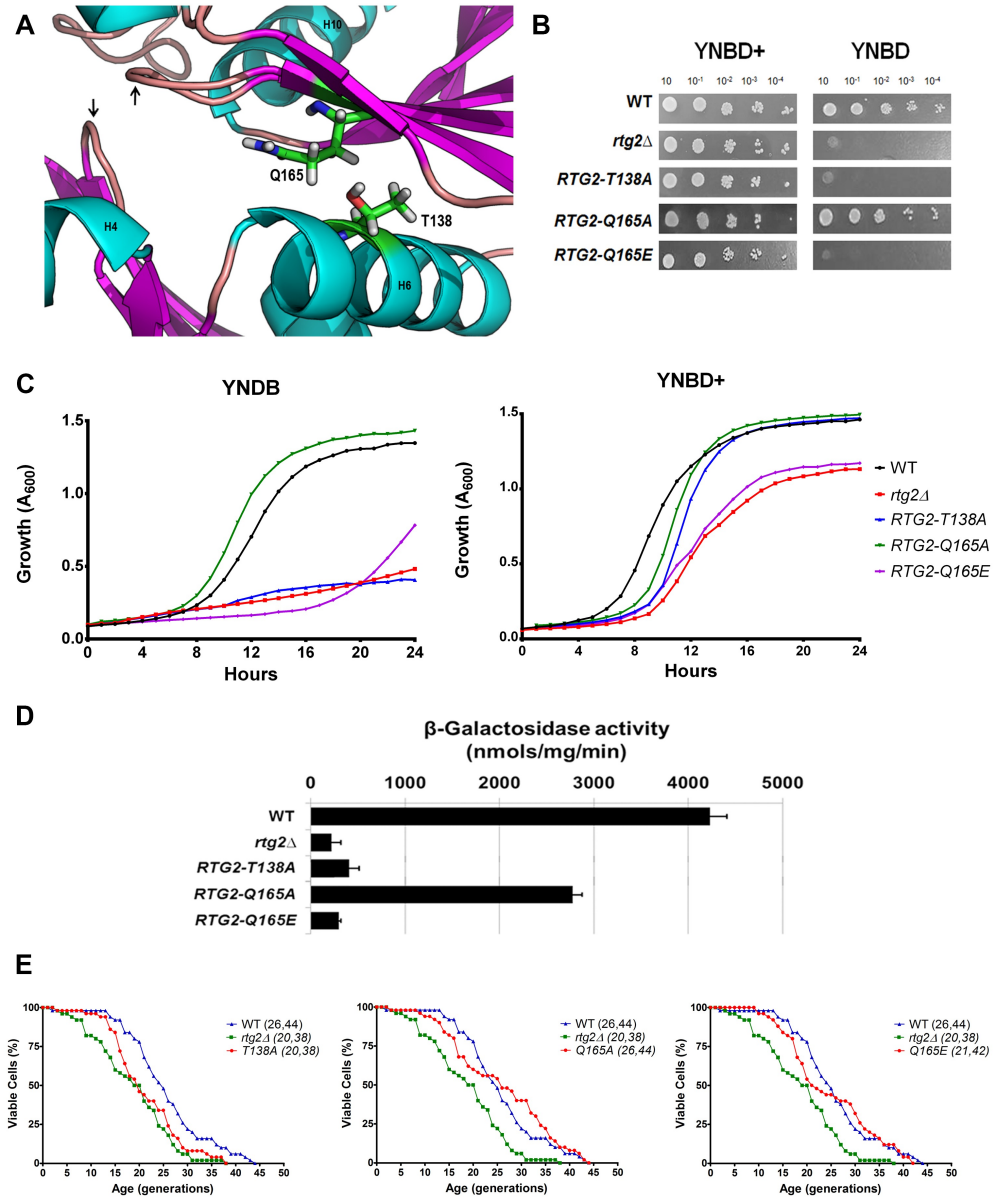
Interaction of coevolved residues T138 and Q165 determine Rtg2p function. **(A)** Location of T138 and Q165 residues on Rtg2p protein structural model. ATP-binding loops are indicated with arrows. **(B)** *RTG2-T138A*, *RTG-Q165E* strains but not *RTG2-Q165A* are glutamate auxotrophs. Glutamate auxotrophy assays were performed by spotting serial dilutions of cells on solid minimal media YNBD and YNBD+ (YNBD plus glutamate at 0.02%). **(C)** Growth performance of strains on minimal liquid medium. Wild type, *rtg2*Δ and mutants *RTG2-T138A*, *RTG-Q165E*, and *RTG2-Q165A* strains were grown on YPD until saturation, diluted to A_600_ = 0.1 in 200 μL of either YNBD or YBND+, and the growth was monitored in a INFINITY PRO 200 (Tecan) microplate reader as described in Materials and Methods section. **(D)** *RTG2-Q165A* strain that mimics T138 and Q165 interaction shows 65% of retrograde signaling activation on minimal medium. Wild type, *rtg2*Δ and point mutant strains *RTG2-T138A*, *RTG-Q165E*, and *RTG2-Q165A* were grown on YNBD medium until A_600_ = 0.6 and cell extracts were prepared to analyze *CIT2-LacZ* expression. β-galactosidase assays were performed in triplicate as previously described (see Materials and methods) and the activity normalized by total protein concentration. **(E)** Mutations *RTG2-T138A* (left panel) and *RTG-Q165E* (right panel) but not *RTG-Q165A* (middle, panel) show short lifespan. In at least two independent experiments, fifty cells of each strains were aligned on YPD and daughter cells were removed from mothers to construct survival curves. The mean and maximum longevity are indicated between parentheses (mean, maximum). Statistical significance between samples is summarized in Table 2.

The amino acids alanine and glutamate in positions 138 and 165 were found to be a coevolved pair in community 3 of our DRCN analysis of the Pfam family PF02541; and they are present in the majority of the sequences of the filtered alignment. However, Rtg2p contains threonine and glutamine in equivalent positions of the family (Table 1). By inspection of Rtg2p model structure we proposed that this divergence would be due to a necessary hydrogen bonding interaction between these two residues (Fig 3A), that is relevant for the protein function. To disrupt this interaction we generated the mutant *RTG2-T138A* that showed glutamate auxotrophy in both solid and liquid medium (Fig 3B and 3C), a very low *CIT2* expression (Fig 3D), and decreased longevity in YPD (Fig 3E, left panel), all of which phenotypes comparable to *rtg2*Δ strain, that corroborated the hypothesis. Similar phenotypes were observed in the *RTG2-Q165E* mutant (Fig 3B–3D and 3E, right panel) The interaction between T138 and Q165 could be simulated by constructing the mutant *RTG2-Q165A*. We expected that this mutant would behave just like wild type cells because the hydrophobic interaction between the alanine residue and the methyl group of threonine would take place to stabilize Rtg2p structure locally. *RTG2-Q165A* cells are glutamate prototrophs, activate *CIT2-LacZ* gene expression (at 65% of the wild type levels) and show similar longevity to the wild type parent (Fig 3B–3D and 3E, middle panel). These results strongly indicate that T138 located at helix 6 and Q165 of strand 7 of the modeled structure may undergo a hydrogen bonding interaction required for Rtg2p protein function and reinforce their importance in the community 3 of coevolved residues.

### Rtg2p functions are impaired by mutations in surface residues E106, R109 but not N113

**Fig 4:**
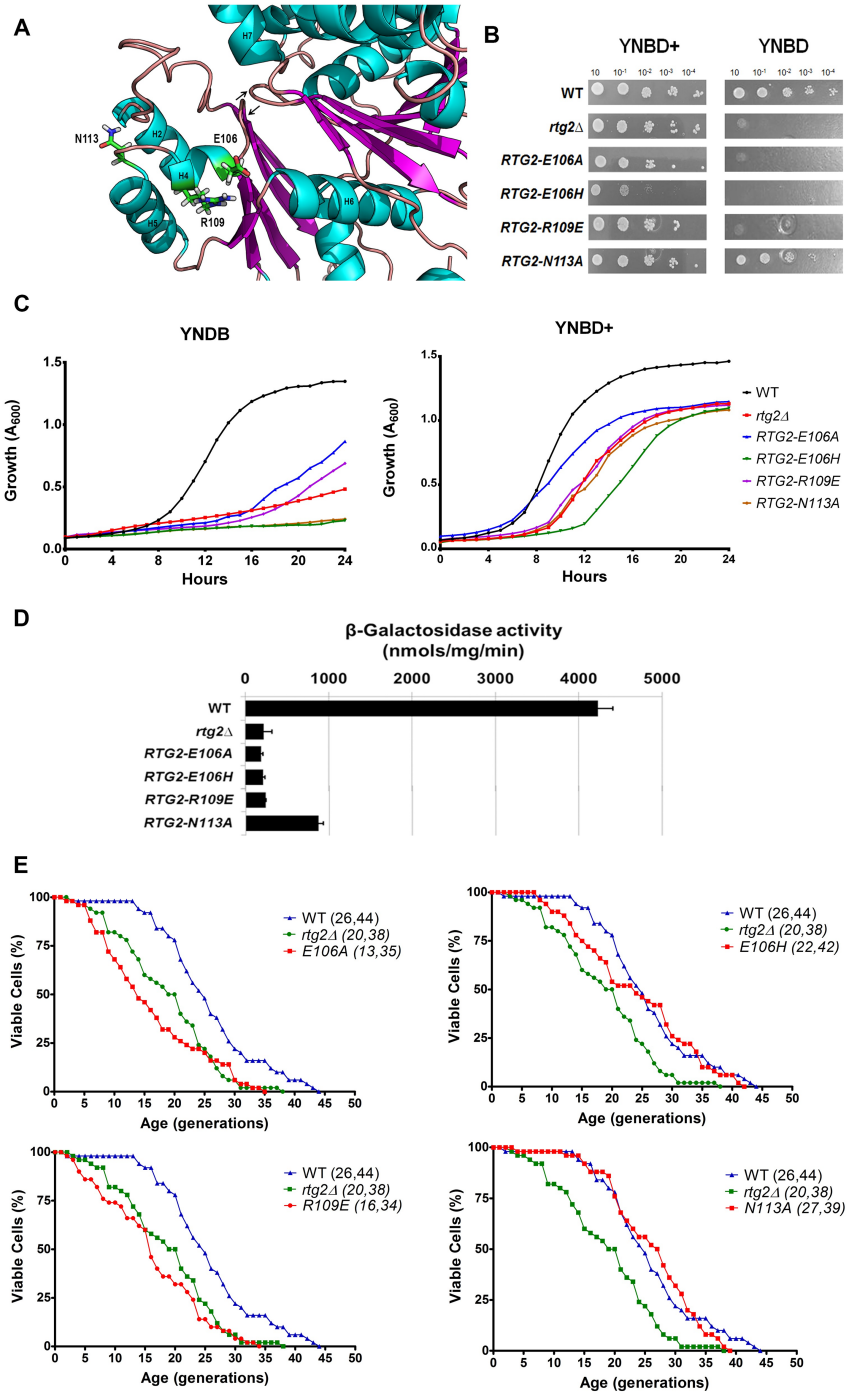
Mutations in Rtg2p surface residues E106, R109 and N113 impair protein function. **(A)** Location of E106, R109 and N113 residues on Rtg2p protein structural model. ATP-binding loops are indicated with arrows. **(B)** Mutant strains *RTG2-E106A*, *RTG2-E106H*, *RTG-R109E* but not *RTG2-N113A* are auxotrophic for glutamate. Auxotrophy assays were performed on solid minimal media YNBD and YNBD+ (YNBD plus glutamate at 0.02%) by spotting serial dilutions of cells that were incubated at 30°C for three days. **(C)** Growth performance of strains on minimal liquid medium. Wild type, *rtg2*Δ and point mutants strains were grown on YPD until saturation, diluted to A_600_ = 0.1 in 200 μL of either YNBD or YBND+, and growth was monitored as described in Materials and Methods. **(D)** *RTG2-N113A* mutant strain retains 21% of wild type *CIT2-LacZ* expression in minimal medium. Wild type, *rtg2*Δ and point mutants were grown on YNBD medium until A_600_ = 0.6 and cell extracts were prepared to analyze *CIT2-LacZ* expression. Triplicate β-galactosidase assays were performed and the activity normalized by total protein concentration. **(E)** Longevity assays show that lifespan of mutant strain *RTG2-E106A* (top left panel) is significantly reduced, which is not observed in *RTG2-E106H* (top right panel), *RTG-R109E* (bottom left panel), and *RTG2-N113A* (bottom right panel) strains. Fifty cells of each strain were aligned on YPD and daughter cells were removed from mothers to construct survival curves from at least two independent experiments. The mean and maximum longevity are indicated between parentheses (mean, maximum). Statistical significance between samples is summarized in Table 2.

A set of conserved residues identified in our analysis are located at the surface of Rtg2p. E106 and R109 are located in a small 5 residue α helix between β4 and H5 (Fig 4A) and may be involved in polar contacts with one another to stabilize its helix turn. To test if this interaction is required for Rtg2p function, we generated the mutant E106A to remove the negative charge of this residue. As expected, *RTG2-E106A* mutant showed glutamate auxotrophy in both solid and liquid media (Fig 4B and 4C), *CIT2* expression similar to the *rtg2*Δ strain (Fig 4D), and reduced RLS in YPD when compared to the *rtg2*Δ strain (Fig 4E, top left panel). When R109 was substituted by glutamate in *RTG2-R109E* mutant, the residue charge was changed to negative to promote repulsion with E106 residue charge, and the cells showed similar phenotypes as observed in *RTG2-E106A* mutant (Fig 4B–4D and 4E, bottom left panel). Likewise, in *RTG2-E106H* mutant, a possible charge repulsion with R109 resulted in cells auxotrophic for glutamate, unable to activate retrograde signaling and *CIT2* transcription but with replicative longevity similar to that of wild type strain (Fig 4B–4D and 4E, top right panel). We speculate that the small helix in which E106 and R109 are located at the periphery of Rtg2p may interact with some other proteins or help Rtg2p form oligomeric structures [10].

Although these results were anticipated from our hypothesis, the mutant *RTG2-E106H* also showed a poor growth even in the presence of glutamate, suggesting an important role for cell viability. In the case of *RTG2-N113A* mutant, we also found unexpected results such as glutamate prototrophy in solid medium, a very long lag phase in liquid YNBD, *CIT2* expression at 21% of wild type levels and no difference from the parent strain regarding to replicative longevity (Fig 4B–4D and 4E, bottom right panel). Altogether these results corroborate the possibility of interaction between E106 and R109 in Rtg2p small helix formed by residues 106–110, and indicates that the conserved residues may be relevant for some but not all functions of the protein, that may include augmenting viability.

### Residues A160 and G161 of Rtg2p ATP-binding loop are involved in retrograde signaling but are not determinants of yeast longevity

**Fig 5:**
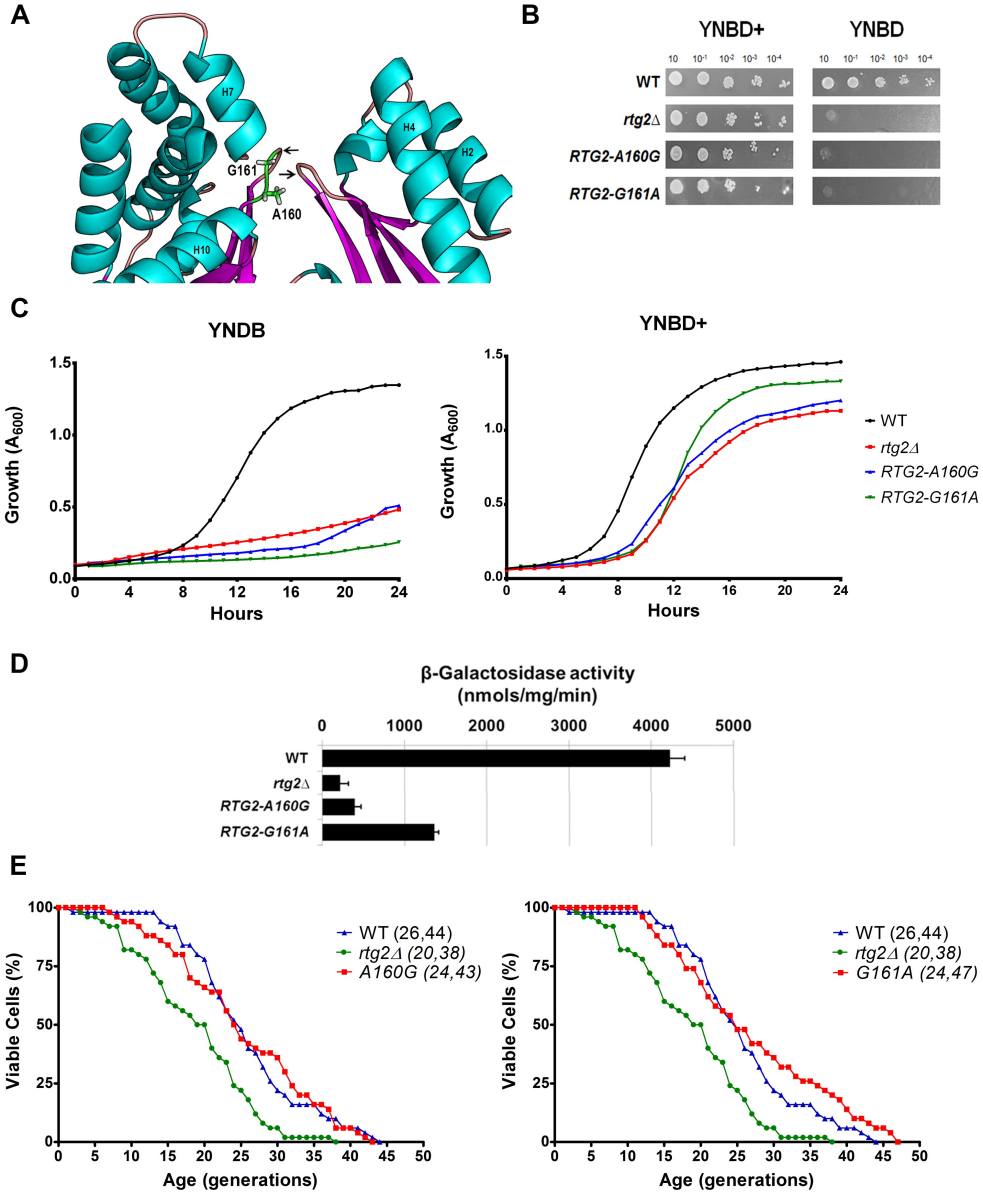
Mutation on residues A160 and G161 of Rtg2p ATP-binding loop decreases retrograde signaling, but not affect yeast longevity. **(A)** Location of A160 and G161 residues on Rtg2p protein structural model. ATP-binding loops are indicated with arrows. **(B)** Both *RTG2-A160G* and *RTG2-G161A* strains show glutamate auxotrophy. Cells were grown on YPD to A_600_ = 1 and four serial dilutions were spotted on solid minimal media YNBD and YNBD+ (YNBD plus glutamate at 0.02%). The plates were incubated at 30°C for three days when photographs were taken. **(C)** Growth performance of mutant strains on minimal liquid medium are similar to that of *rtg2*Δ strain. Wild type, *rtg2*Δ and point mutants strains were grown on YPD until saturation, and diluted to A_600_ = 0.1 in either YNBD or YBND+, and growth curves were obtained in a INFINITY PRO 200 (Tecan) microplate reader, as described in Materials and Methods. **(D)** Expression of *CIT2-LacZ* reporter of the *RTG2-G161A* mutant is 32% of wild type parent expression. Wild type, *rtg2*Δ and point mutants were grown on YNBD medium, and cell extracts were prepared to analyze *CIT2-LacZ* expression. β-galactosidase assays were performed in triplicate and the activity normalized by total protein concentration. **(E)** Replicative lifespan of both mutant strains *RTG2-A160G* (left panel) and *RTG2-G161A* (right panel) are similar to WT. Fifty cells of each strain were aligned on YPD and daughter cells were removed from mothers to construct survival curves. The mean and maximum longevity are indicated between parentheses (mean, maximum). Statistical significance are summarized in Table 2.

The active site of proteins in the ASKHA family is located in a cleft that also divides Rtg2p N-terminal domain. In this cleft, conserved loop structures are involved in ATP binding [43]. The loop between strands β6 and β7 contains an imperfect (D/N)XG motif characteristic of ASKHA phosphotransferases [29] and its side chain residues (D158, V159, A160, G161, G162 and S163) are possibly critical for phosphate binding. In our analysis we identified Rtg2p A160 as a divergent residue from the rest of family members, that contains a glycine in 99.8% of the sequences in an equivalent position (S4 Table). To investigate the reason of this difference we generated the mutant *RTG2-A160G*, and also *RTG2-G161A* given the strong conservation for G161 found in our analysis. These mutant strains showed glutamate auxotrophy in both solid and liquid media (Fig 5B and 5C), and *RTG2-A160G* was blocked in reporter gene expression whereas *RTG2-G161A* strain retained 32% of *CIT2* expression observed in the wild type control (Fig 5D); and although the mutation G161A had an slightly difference in the longevity curve (Fig 5E, right panel), it was not significant (Table 2, and also WT<G161A one-tailed test p = 0.1532).

**Table 2:** Statistical analysis of RLS experiments performed in YPD.

Strain	WT	*rtg2*Δ
WT	ns	**
*rtg2*Δ	**	ns
*L56G*	**	**
*E106A*	**	ns
*E106H*	ns	**
*R109E*	**	ns
*N113A*	ns	**
*E137A*	**	ns
*T138A*	**	ns
*D158A*	**	ns
*A160G*	ns	**
*G161A*	ns	**
*S163A*	ns	**
*Q165A*	ns	**
*Q165E*	ns	**

P-values were determined by Mann-Whitney test. Hypothesis test: WT column: mutant ≠ WT; *rtg2*Δ column: mutant ≠ *rtg2*Δ. ns: not significant.

** p <0.0001.

Collectively these results indicate that residues A160 and G161 are involved in the transmission of retrograde signaling, and have longevity in YPD comparable to the wild type strain when mutated, respectively, to glycine and alanine. In addition, although all ASKHA family members have glycine in the equivalent position of Rtg2p A160, this divergent residue is crucial for protein function in the later.

### E137, D158, and S163 are probably residues involved in coordination of Mg^2+^ ion in the ATP binding site of Rtg2p

**Fig 6:**
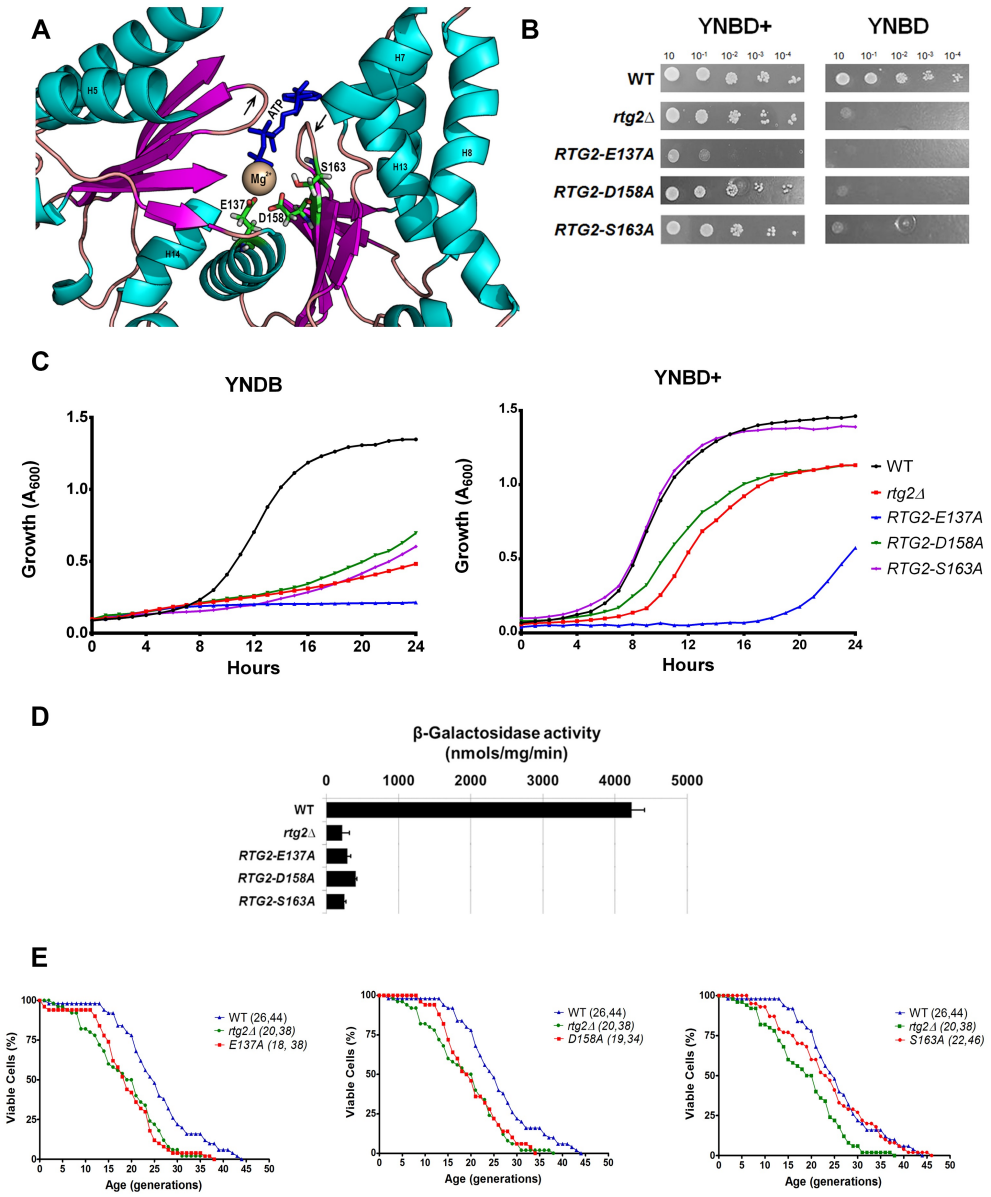
E137, D158, and S163 residues are likely involved in the coordination of Mg^2+^ ion at the ATP binding site of Rtg2p. **(A)** Location of E137, D158, and S163 residues on ATP binding site of Rtg2p protein structural model. ATP-binding loops are indicated with arrows. **(B)** Mutations E137A, D158A, and S163A on Rtg2p cause glutamate auxotrophy to yeast cells. Glutamate auxotrophy assays were performed by spotting serial dilutions of cells on solid minimal media YNBD and YNBD+ (YNBD plus glutamate at 0.02%). **(C)** Growth performance of mutant strains on YNBD liquid medium are similar to that of *rtg2*Δ strain. Wild type, *rtg2*Δ and *RTG2-E137A*, *RTG2-D158A*, and *RTG2-S163A* strains were grown on YPD until saturation, diluted to A_600_ = 0.1 in either YNBD or YBND+, and incubated at 30°C, 160 rpm, for 24 h to monitor growth in a INFINITY PRO 200 (Tecan) microplate reader. **(D)** Retrograde signaling is not activated in *RTG2-E137A*, *RTG2-D158A*, and *RTG2-S163A* mutant strains. Wild type, *rtg2*Δ and point mutants were grown on YNBD medium until A_600_ = 0.6 and cell extracts were prepared to analyze *CIT2-LacZ* expression. β-galactosidase assays were performed as previously described (see Materials and Methods), and the activity normalized by total protein concentration. **(E)** Rtg2p mutations E137A (left panel) and D158A (middle panel) but not S163A (right panel) decrease the replicative lifespan of yeast. Fifty cells of each strain were aligned on YPD and daughter cells were removed from mothers to construct survival curves from at least two independent experiments. The mean and maximum lifespan are indicated between parentheses (mean, maximum). Statistical significance are summarized in Table 2.

Previous work established the residues involved in Mg^2+^ ion coordination in the ATP-biding site of ASKHA family members. The two loop structures with (D/N)XG motif contribute with aspartate residues that act as ligands through water molecules that coordinate magnesium in an octahedral arrangement [20]. In agreement with that, our conserved residue analysis pointed to D158, that is located at the loop between strands β6 and β7, as a conserved residue important for Rtg2p function (Fig 6A). Moreover, it also found S163 located in the same loop and E137, a residue of helix 6 that is present in 100% of the sequences in the alignment (Fig 6A), as good candidates for mutagenesis and phenotypical analysis. To investigate the contribution of these residues to Rtg2p function we constructed the alanine mutants *RTG2-D158A*, *RTG2-S163A*, and *RTG2-E137A*, all of which resulted in cells auxotrophic for glutamate, blocked in retrograde signaling, but only *RTG2-D158A* and *RTG2-E137A* showed reduced longevity in YPD rich medium (Fig 6B–6E). The strain *RTG2-S163A* showed no difference in lifespan when compared to wild type cells (Fig 6E, left panel).

Given the structural similarity of Rtg2p with other members of the ASKHA protein family, these data strongly suggest that residues D158, E137 and S163 are probably involved in polar interaction with water molecules that may coordinate Mg^2+^ ion in the active site of the protein. It also worth to mention that cells *RTG2-E137A* grew poorly even in the presence of glutamate (Fig 6B and 6C), a phenotype also observed in *RTG2-E106H* mutant (Fig 4B and 4C), that indicates this residue is important for cell viability.

### Some *RTG2* point mutations extend the lifespan of PSY142 ρ^0^ cells grown in raffinose

Northern blot analyses of *CIT2* expression revealed that in some strain backgrounds [13], and also in PSY142 [6], glucose represses the transcriptional activation of retrograde signaling. However, the increase in up to 30-fold of *CIT2* expression of ρ^0^ cells compared to that of ρ^+^ parent can be uncovered in raffinose [5, 13], a nonrepressing fermentable carbon source. This difference in carbon source may promote a significant increase in RLS of ρ^0^ cells, such as that observed in SP1-1 strain, whose petite have shorter RLS in glucose and longer RLS in raffinose when compared to the WT ρ^+^ parent [13]. As retrograde signaling is activated in ρ^0^ cells grown in raffinose, and taking into account a possible difference in RLS that could be observed, we decided to address the effect of the carbon source on the expression of *CIT2-LacZ*, and also on the RLS of the thirteen mutants generated.

**Fig 7:**
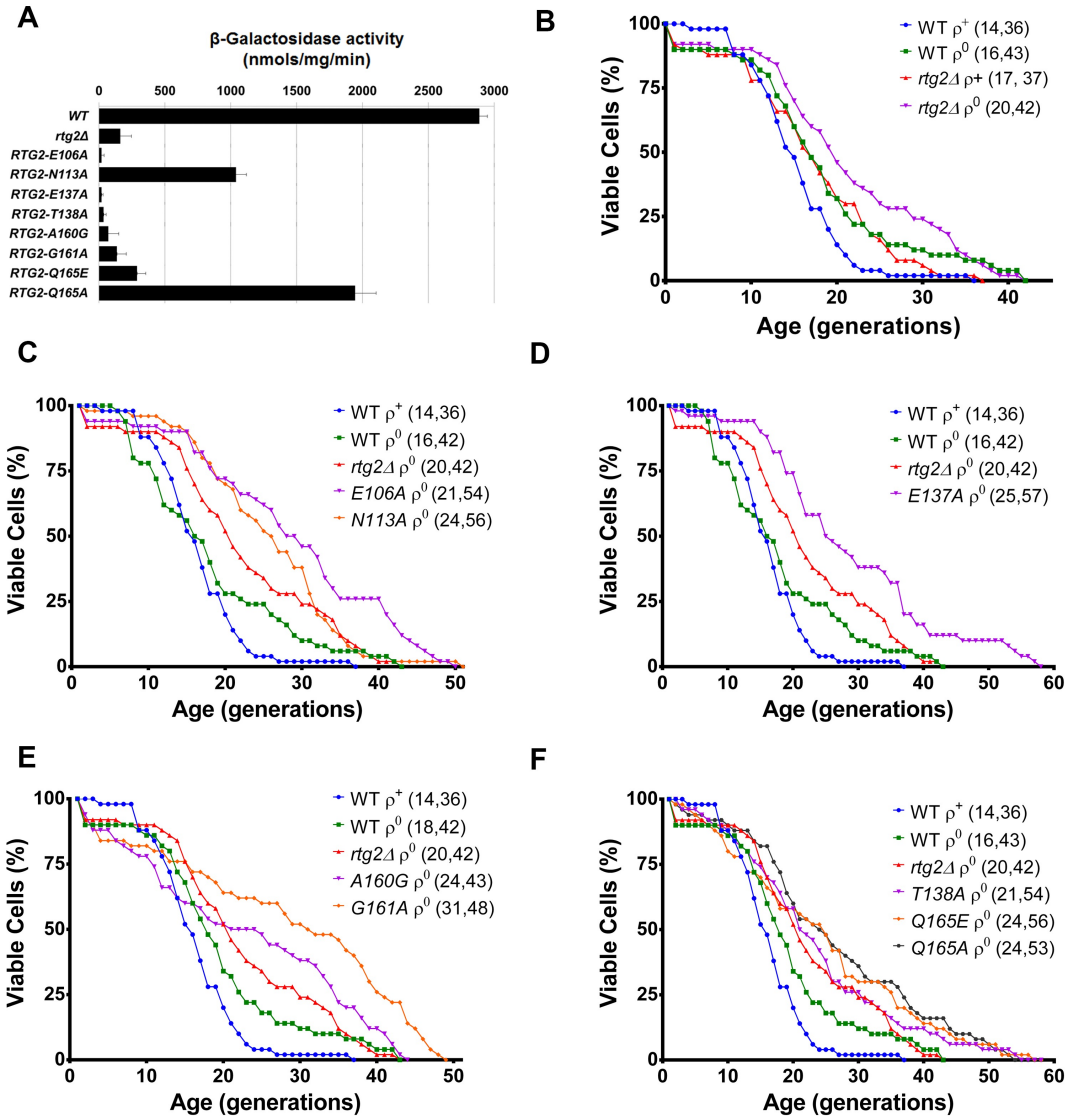
When grown in raffinose, *RTG2* point mutations in ρ^0^ background extend the lifespan when compared to PSY142 ρ^0^. **(A)** *RTG2-N113A* and *RTG2-Q165A* mutants retain partial *CIT2* expression. Petite strains, generated from wild type, *rtg2*Δ and *RTG2* point mutants, were grown on YPR medium until A_600_ = 0.6, and cell extracts were prepared to analyze *CIT2-LacZ* expression. β-galactosidase assays were performed in triplicate as described in the Materials and Methods section and the activity normalized by total protein concentration. **(B)** PSY142 ρ^0^ strain shows extended RLS when compared to its ρ^+^ parent and no difference when compared to *rtg2*Δ ρ^0^. **(C)** *RTG2-E106A* ρ^0^ and *RTG2-N113A* ρ^0^ strains show enhanced longevity increase in RLS compared to WT ρ^0^. **(D)** *RTG2-E137A*, a residue involved in Mg^2+^ ion coordination, have significantly extended lifespan in ρ^0^ background if compared to WT ρ^0^. **(E)** The strain *RTG2-G161A* ρ^0^ but not *RTG2A160G* ρ^0^ robustly increases RLS when compared to WT ρ^0^ **(F)** Both strains *RTG2-Q165E* ρ^0^ and *RTG2-Q165A* ρ^0^ significantly increased RLS, but the mutant *RTG2-T138A* ρ^0^ is similar to WT ρ^0^. In replicative longevity assays, fifty cells of each strain were aligned on YPR and daughter cells were removed from mothers to construct survival curves from at least two independent experiments. The mean and maximum longevity are indicated between parentheses (mean, maximum). Statistical significance between samples is summarized in Table 3.

In raffinose, when compared to the ρ^+^, the lifespan of the PSY142 ρ^0^ strain is extended (Fig 7B), but the increase in RLS is not dependent on *RTG2*, because there is no significant difference in longevity between ρ^0^ and *rtg2*Δ ρ^0^ strains. A similar observation is made with *RTG2-A160G* and *RTG2-T138A* (Fig 7E and 7F), and in *RTG2-L56G* (S5B Fig), *RTG2-E106H*, *RTG2-R109E* (S5C Fig), *RTG2-D158A* (S5D Fig), and *RTG2-S163A* (S5E Fig) point mutations in ρ^0^ background. The expression of *CIT2-LacZ* in these mutants was also marginal (Fig 7A and S5A Fig). Nevertheless, some of the *RTG2* point mutants showed a robust enhance in RLS compared to WT ρ^0^, as in *RTG2-E106A* ρ^0^ (Fig 7C), *RTG2-E137A* ρ^0^ (Fig 7D), *RTG2-G161A* ρ^0^ (Fig 7E), *RTG2-Q165E* ρ^0^ (Fig 7F) without significant activation of *CIT2* transcription, whereas *RTG2-N113A* ρ^0^ (Fig 7C), and *RTG2-Q165A* ρ^0^. (Fig 7F) extended RLS and also showed, respectively, 36 and 67% of the expression observed in *RTG2* ρ^0^.

**Table 3:** Statistical analysis of RLS experiments conducted in YPR.

Strains	WT	WT ρ^0^	*rtg2*Δ ρ^0^
WT	ns	*	***
WT ρ^0^	*	ns	ns
*rtg2*Δ	ns	ns	*
*rtg2*Δ ρ^0^	***	ns	ns
*L56G* ρ^0^	*	ns	ns
*E106A* ρ^0^	***	**	*
*E106H* ρ^0^	ns	ns	***
*R109E* ρ^0^	ns	ns	ns
*N113A* ρ^0^	***	***	*
*E137A* ρ^0^	***	***	**
*T138A* ρ^0^	***	ns	ns
*D158A* ρ^0^	ns	ns	*
*A160G* ρ^0^	*	ns	ns
*G161A* ρ^0^	***	**	*
*S163A* ρ^0^	*	ns	ns
*Q165A* ρ^0^	***	**	*
*Q165E* ρ^0^	***	*	ns

P-values were determined by Mann-Whitney test. Hypothesis test: WT column: mutant ≠ WT; WT ρ^0^ column: mutant ≠ WT ρ^0^. *rtg2*Δ ρ^0^ column: mutant ≠ *rtg2*Δ ρ^0^. ns: not significant.

* p <0.05,

** p <0.01,

*** p <0.001.

These observations suggest that lifespan extension of PSY142 ρ^0^ is dependent on carbon source and independent on *RTG2* gene, as illustrated by a complete gene disruption or by some *RTG2* point mutants. They suggest further that retrograde signaling is not required for lifespan extension in ρ^0^ cells grown in raffinose, and that retrograde response activation and RLS extension are not mutually exclusive, as show in *RTG2-N113A* ρ^0^ and *RTG2-Q165A* ρ^0^ mutants that retained partial activation of *CIT2* transcription.

## Discussion

Retrograde signaling, a pathway of communication from mitochondria to the nucleus, activates genes whose transcription is either independent or dependent on the action of Rtg1/3p transcription factor complex and Rtg2p protein (RTG pathway). Three conditions can activate the RTG pathway: ρ^0^ cells grown in rich or minimal medium with raffinose [1, 6], ρ^+^ cells grown in minimal medium under glutamate starvation (YNBD medium) [5], and ρ^+^ cells treated with the macrolide rapamycin, that inhibits the TOR pathway [9, 44]. Rtg2p is a key positive modulator required to activate retrograde signaling in response to such conditions in yeast. This activation is dependent on its N-terminal putative ATP-binding domain that is a member of the ASKHA superfamily of phosphotransferases. Although there is a wealth of information about this family, to date there is no extensive structural characterization of Rtg2p. In the present work, we performed residue conservation and correlation analysis, and generated thirteen point mutants by site-directed mutagenesis, to identify the determinants of Rtg2p in retrograde signaling and longevity. We constructed a 3D molecular model to locate the mutated residues on Rtg2p structure. This model allowed us to generate hypotheses that were tested to understand the contribution of the individual residues to the protein function. The majority of mutated residues affected the functions of the protein rendering cells auxotrophic for glutamate, unable to activate *CIT2* gene expression and with *rtg2*Δ, wild type or long-lived phenotypes regarding to lifespan. This phenotype analysis together with our Rtg2p model located the residues of the protein required for its function in retrograde signaling (RTG pathway) and longevity.

*CIT2-LacZ* expression and glutamate auxotrophy were surrogate phenotypes used to study the function of Rtg2p in both metabolic reprogramming and retrograde response. Although the majority of mutants that are glutamate auxotrophs have decreased lifespan, it is important to mention that glutamate auxotrophy does not cause lifespan changes *per se*, as clearly observed in the mutant *RTG2-L56G*, that is auxotrophic for glutamate but showed an extension in lifespan in YPD. In addition, *CIT2* expression is not a requirement for yeast longevity, as shown previously in two distinct genetic backgrounds, YPK9 and YSK365 [13]. Finally, the ability of the mutants to grow in rich medium YPD or YPR is not compromised by either *RTG2* deletion or point mutation (S4 Fig), except for a mild effect observed in *RTG2-E106H* (S4A Fig).

Previous work showed an alignment of amino acid sequences of the GppA and Ppx proteins that included *S*. *cerevisiae* Rtg2p, and this analysis revealed the conservation of five motifs, related to the binding of ATP (PHOSPHATE 1, PHOSPHATE 2, CONNECT 1, CONNECT 2, and ADENOSINE) [19]. The conservation of such motifs suggests that the structural core of the family is present in Rtg2p. Indeed, Liu and co-workers have shown that mutations in the motifs PHOSPHATE 2 (G154D), ADENOSINE (G266V), and CONNECT 2 (G311D) of Rtg2p generated cells with reduced *CIT2* expression and decreased ability of Rtg2p to bind the negative regulator Mks1p [9]. The residues found in the DRCN conservation analysis were also located in these motifs, E137 and T138 in CONNECT 1 and D158, A160, G161, S163, and Q165 (that coevolved with T138 in community 3, Fig 1B) in PHOSPHATE 2. Whereas most of this residues when mutated impaired retrograde signaling in YPD, Q165A and G161A mutations retained part of this activation as shown by *CIT2* expression (respectively, Figs 3D and 5D). When Q165 is mutated to glutamate *CIT2* expression level is similar to that of *rtg2*Δ strain. Therefore, the residues conserved in the N-terminus of Rtg2p, present in two out of five conserved ASKHA family motifs, depending on the chemical environment modification, may not impair retrograde signaling.

Remarkably, L56 is a conserved Rtg2p residue, that is not present in any of the five motifs related to ATP-binding, whose mutation to glycine confers the most dramatic extension in mean and maximum longevity in YPD rich medium (Fig 2E) with no activation of retrograde signaling (Fig 2B–2D). This is completely unexpected because most of mutations in Rtg2p N-terminal domain render cells with lifespan similar to *rtg2*Δ phenotype with pronounced decreased in longevity when compared to wild type parent. This mutant clearly shows there is a separation between retrograde signaling pathway and lifespan extension functions in Rtg2p. Indeed, one of the functions of Rtg2p is the suppression of extrachromosomal rDNA circles (ERCs) that accumulate in yeast cells as they age [16, 17]. In petite mutants that lack mitochondrial DNA the retrograde signaling is active and such cells accumulate high levels of ERCs [18]. This suggested that when involved in the induction of retrograde response Rtg2p is not able to counter ERC accumulation which causes cell death [16]. Therefore, the mutation L56G inactivated retrograde signaling but increased lifespan, possibly augmenting the interaction of Rtg2p with other proteins, and such complexes are able to inhibit ERC accumulation and promote longevity extension.

Among Rtg2p conserved residues found in DRCN analysis E137 (CONNECT 1), D158 and Q165 (PHOSPHATE 2) are also conserved in *Escherichia coli* exopolyphosphatase (equivalent residues E121, D143, and E150). *E*. *coli* Ppx is a protein that degrades long chains of ionorganic polyphosphate [29, 45, 46]. Mutations in its equivalent residues diminished the exopolyphosphatase activity to a residual level [29]. In Rtg2p, mutation in D158 generated a strain with very low *CIT2* expression (Fig 6D), and mutation in E137 gave rise to cells with defects in both retrograde signaling and longevity maintenance (Fig 6D and 6E, left panel). The mutation Q165E produced a glutamate auxotroph strain, but the mutation Q165A grows as well as the wild type in YNBD without glutamate (Fig 3B and 3C). Both *RTG2-Q165A* and *RTG2-Q165E* mutants have similar lifespans, that are presumably due to movement restrain of a protein hinge (discussed below) and an increase in the amount of residues available to chelate magnesium ion in the ATP-binding site, respectively. Indeed, the mutation Q165E is in the vicinity of E137 and D158, that are located in positions know to be involved in Mg^2+^ biding and stabilization of ATP at the active site, in other members of the ASKHA protein family. Rtg2p is the only member of its family that contains a glutamine in this position (Q165) whereas all the others contain glutamate at the equivalent position, such as *E*. *coli* Ppx exopolyphosphatase (E150). This residue is critical for ATP binding of exopolyphosphatases and is commonly found in members of the ASKHA family, but *S*. *cerevisiae* found an adaptive advantage in Q165 for activating of retrograde signaling.

The community 3 of amino acids present in Rtg2p (T138 and Q165) diverge from those found in our analysis of the Pfam family PF02541 (alanine and glutamate on equivalent positions). After locating these residues in our Rtg2p structural model, we hypothesized that this divergence would be due to a hydrogen bonding interaction between these two residues (Fig 3A), that could be significant for protein function. Disruption of this interaction confirmed this hypothesis because the mutant *RTG2-T138A* showed glutamate auxotrophy (Fig 3B and 3C), diminished *CIT2* expression (Fig 3D), and reduction of longevity in YPD (Fig 3E, left panel), phenotypes comparable to those of *rtg2*Δ strain. We also simulated the interaction between T138 and Q165 in the mutant *RTG2-Q165A*, that behaved just like wild type cells, because the hydrophobic interaction between the alanine residue and the methyl group of threonine probably stabilized Rtg2p locally in a similar way (Fig 3B–3D and 3E, middle panel). The interaction between T138 and Q165 in Rtg2p associates the helix 6 that contain residues of CONNECT1 with strand seven that belongs to the PHOSHATE2 motif. This restraint caused by Q165 is absent in all known structures of the family, that contain glutamate in the equivalent position, and, for this reason, are more mobile. In the ASKHA superfamily domain movements up to 30° required for catalysis are observed [43]. One example is the crystal structure of *Aquifex aeolicus* Ppx/GppA [47], that was studied from two crystals forms type I and type II, that revealed the protein has a structural flexibility previously described as "butterfly-like" cleft opening in other members of the family [48]. The domain configuration of these structures indicated the residues Y122 and A123 as a protein hinge region that allowed a rotational flexibility of 11.5° [47]. In Rtg2p, the equivalent residues are V140 and G141, and just beside this putative hinge lies the residue T138 that may interact with Q165, possibly to prevent the "butterfly-like" movement around the active site of the protein. We expect this restriction in mobility may result in a better proximity between the two ATP-binding loops of the protein, that are required for Rtg2p function. This exclusive characteristic of Rtg2p identified in the coevolved pair T138-Q165 indicates an evolutionary advantage, as the mutants *RTG2-T138A* and *RTG2-Q165E* that contain exactly the residues that coevolved in the family are not functional regarding the phenotypes analyzed in YNBD and YPD.

ATP binding and hydrolysis depend on the presence of Mg^2+^ and/or Mn^2+^ ions, is already found in structures of the members of the ASKHA superfamily such as Hsc70 [20]. Solvent exposed Mg^2+^ ions have a critical role in the active site of Hsc70 mediating the binding of ATP. In hexokinases, Mg^2+^ ions are coordinated by an aspartate, in Hsc70 and actin glutamate and glutamine participate of nucleotide binding [20, 29]. Given the importance of Mg^2+^ ions, our Rtg2p model was submitted to the server 3DLigandSite [49] that indicated residues with similar interaction with Mg^2+^ ions to stabilize ATP binding. Based in this structure, the conserved residues found by DRCN analysis and the known residues of the superfamily involved in this binding, we identified E137, D158 and S163 as three probable residues that may facilitate the binding of ATP in the active site of Rtg2p (Fig 6A). In this region we mutated individually these residues to alanine to prevent they act as ligands through water molecules to coordinate Mg^2+^ ion in an octahedral arrangement [20]. These mutations decrease dramatically the retrograde signaling to the levels observed in cells lacking *RTG2* (Fig 6B–6D). These results together with the observation that physiological concentration of ATP dissociates the negative regulator of retrograde response Msk1p from Rtg2p [39] strengths the notion that the N-terminal of Rtg2p binds ATP, and we mapped the residues that may be involved in this interaction.

It was unexpected that *RTG2-E137A* and *RTG2-E106H* mutant strains grow poorly in minimal medium even in the presence of glutamate (Fig 6B). However, the strain *RTG2-E106H* showed no significant difference in lifespan from the wild type (Fig 4E, top right panel) whereas the strain *RTG2-E137A* showed a decrease longevity (Fig 6E, right panel). A possible structural explanation for this behavior is the fact that E137 is a residue involved in Mg^2+^ ion binding that is required for the function of Rtg2p involved in longevity. Similarly, D158 is also involved in magnesium binding and the mutant *RTG2-D158A* have decreased longevity, similar to that of *rtg2*Δ mutant (Fig 6E, middle panel). The residue E106 is not involved in Mg^2+^ ion binding, and it is located at the periphery of Rtg2p, in a small 5 residue α helix between β4 and H5 (Fig 4A). This helix may provide the surface for protein-protein interactions, possibly Rtg2p oligomerization [10] but contacts with other proteins are not excluded. Therefore, lack of appropriate coordination of magnesium ion is more detrimental to yeast longevity than loss of a possible interaction with other proteins at the periphery of Rtg2p.

Glucose represses the retrograde signaling activation and the lifespan extension observed in some ρ^0^ strain backgrounds [13], and it is also known to repress *CIT2* expression in PSY142 ρ^0^ strain [6]. Here we show that glucose curtails the effect of longevity extension in these cells, because, in a nonrepressing carbon source such as raffinose, PSY142 ρ^0^ showed extended RLS compared to the grande parent (Fig 7B). In raffinose, regarding the expression of *CIT2-LacZ*, the mutant strains *RTG2-N113A* ρ^0^ and *RTG2-Q165A* ρ^0^ were the only to retain partial transcriptional activation of *CIT2* (Fig 7A). The lifespan of these mutants (respectively, Fig 7C and 7F) and also that of *RTG2-E106A* ρ^0^ (Fig 7C), *RTG2-E137A* ρ^0^ (Fig 7D), *RTG2-G161A* ρ^0^ (Fig 7E), and *RTG2-Q165E* ρ^0^ (Fig 7F) were significantly extended compared to the WT ρ^0^ strain, whereas the other *RTG2* mutant ρ^0^ strains showed no difference (Fig 7C–7F). These surprising findings show that raffinose extends the RLS of PSY142 ρ^0^ cells whose lifespan can be further extended in strains carrying the aforementioned mutations. The structural basis for these observations is rather complex as the mutations are spread over residues in the surface of the protein (N113 and E106), or located at the community 3 of coevolved amino acids (Q165), or involved in magnesium binding (E137). The production of strains with combinations of these mutations may help to determine if they act cooperatively, and this information may possibly integrate the actions of such mutant residues in a single mechanism. Finally, it is noteworthy that *RTG2* deletion does not decrease the lifespan extension of PSY142 ρ^0^ compared to ρ^+^ cells, indicating a phenotype dependent on the genetic background, because it is contrary to previous observations in YPK9 and SP1-1 strains [13].

**Fig 8:**
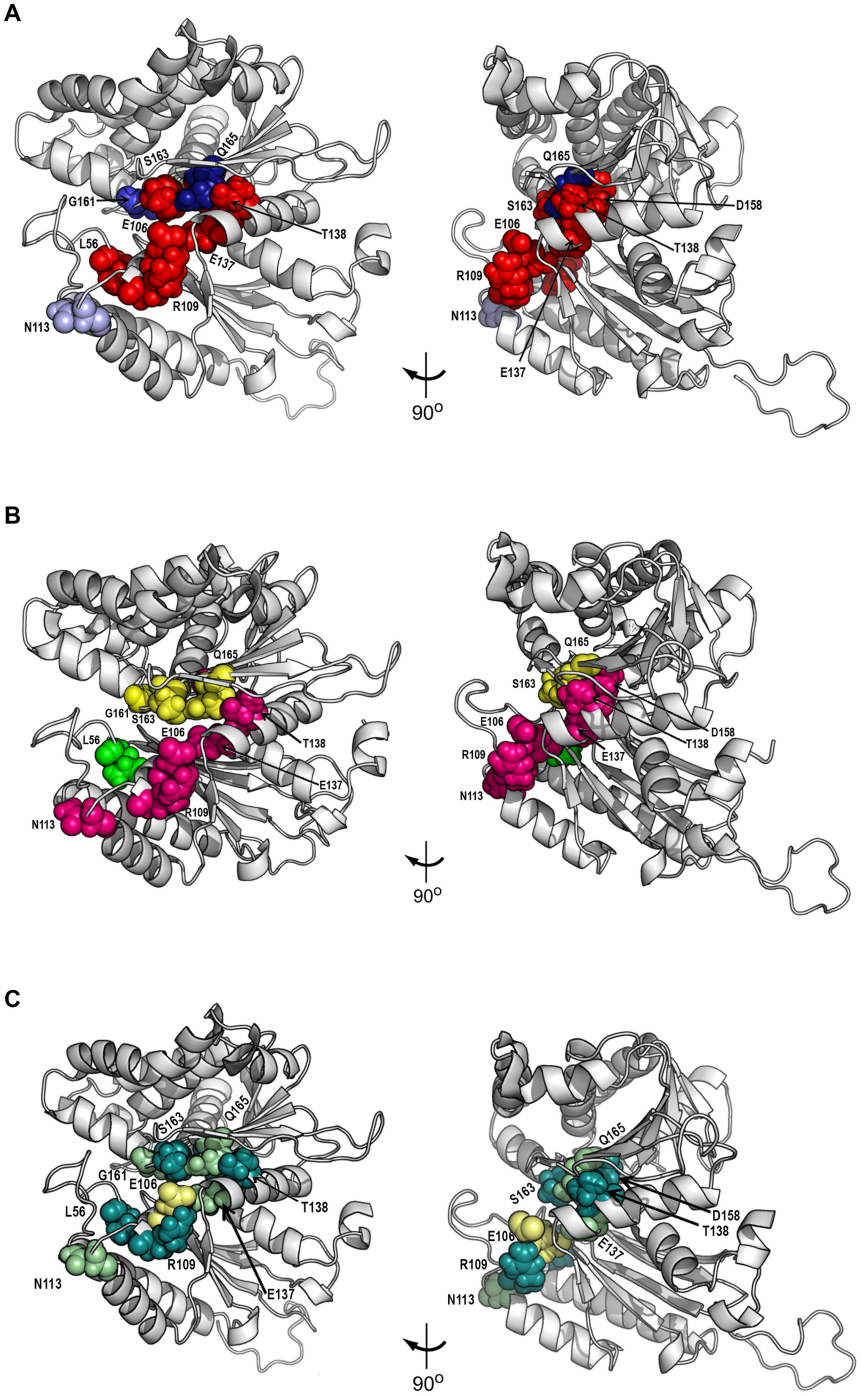
Functional mapping of Rtg2p residues involved in retrograde signaling and aging. **(A)** Retrograde signaling determinants map. This map reflects the gene expression of the reporter *CIT2-LacZ* in the mutant strains grown on YNBD. Red color represents the residues whose β-galactosidase activity was similar to the *rtg2*Δ strain, in which the retrograde response is not activated. Blue color shades indicates the expression observed in *RTG2-N113A*, *RTG2-G161A* and *RTG2-Q165A* mutants that show, respectively 21, 32 and 65% of the wild type expression (the darker blue the higher the level of gene expression). **(B)** Map of Rtg2p lifespan determinants identified with *RTG2* point mutants grown on YPD. N-terminal Rtg2p mutants are divided into three different groups in respect to their longevity phenotype. Mutants showing longevity similar to the wild type are shown in yellow, those similar to *rtg2*Δ in magenta and the long-lived mutants in green. **(C)** Map of Rtg2p lifespan determinants found in *RTG2* ρ^0^ point mutants grown on YPR. N-terminal Rtg2p mutants are divided into two different groups in respect to their longevity phenotype. Mutants showing longevity similar to the WT ρ^0^ are shown in deepteal, those whose RLS was markedly increased are in palegreen; and in paleyellow; the residue E106 whose mutations are in both groups (E106H is long-lived whereas Q165E is short-lived). Residues are displayed in space filling representation and left panels are the same model as right panels rotated 90°clockwise around *y* axis.

Altogether, the phenotypes analyzed of the mutants obtained by site-directed mutagenesis, and the structural 3D model generated, allowed to map on Rtg2p the determinants of retrograde signaling and longevity (Fig 8). Retrograde signaling is mediated by residues that surround Rtg2p N-terminal active site (Fig 8A, red residues). This is in contrast with previous findings whose residues (G154, G266 and G311) map to the opposite side of the domain, away from the active site [9]. These residues were not found in our conservation analysis that excluded positions with conservation below 90% in the filtered alignment. Interestingly, in three positions of Rtg2p it is possible to modulate the activity of the protein such that partial *CIT2-LacZ* expression is observed as in N113A, G161A and Q165A mutants that show, respectively 21, 32 and 65% of the wild type expression (Fig 8A, blue shades). Rtg2p is a protein involved in yeast longevity; in some strain backgrounds, lack of mitochondrial DNA extends lifespan, that is abolished by *rtg2*Δ mutation [13]. Although the lifespan extension in petites is not observed in PSY142 strain grown in YPD (S3 Fig), replicative lifespan assays conducted with our mutants identified the contribution of the individual residues to longevity. When these results are transported to the Rtg2p structural model it is possible to notice the distribution of the residues along the N-terminal domain divides it in three distinct regions (Fig 8B). In the first region are located the residues whose mutation is unable to interfere with Rtg2p function in longevity (E106H, N113A, A160G, G161A, S163A, Q165A, and Q165E) (Fig 8B, yellow surface). The second region contains residues whose mutation rendered cells with aging phenotype similar to *rtg2*Δ strain (E106A, R109E, E137A, T138A, and D158A) (Fig 8B, magenta surface), and the third region contains only the residue L56G, whose mutation produced long-lived cells (Fig 8B, green surface). The functional RLS map of the mutants grown in raffinose also showed three regions; one containing mutated residues that did not further extended the longevity of WT ρ^0^ (L56G, R109E, T138A, D158A, A160G, S163A) (Fig 8C, deepteal), a second region containing residues that significantly increased RLS (N113A, E137A, G161A, and Q165A) (Fig 8C, palegreen), and the residue E106 whose mutations E106H and E106A showed RLS similar to the WT ρ^0^ or an enhanced effect on RLS, respectively (Fig 8C paleyellow). This spatial distribution shows that, apparently, the function of Rtg2p related to longevity reside in a specific part of its N-terminus close to the ATP-binding site. To the best of our knowledge, this the first time the contribution of single Rtg2p residues to yeast aging is demonstrated, and it suggests that this approach could map other functional residues of Rtg2p involved in other pathways within the cell. Moreover, it is also the first report using the DRCN method extensively to map a protein involved in aging.

Recently, the gene *PHO84* was identified as the target of retrograde signaling responsible for lifespan extension in ρ^0^ cells [50]. Pho84p is a high-affinity inorganic phosphate transporter located at the plasma membrane [51, 52] and its expression is decreased in *rtg2*Δ ρ^0^ cells [4]. Although this finding directs the research on lifespan extension induced by retrograde signaling toward the proximal effector of longevity downstream of *PHO84*, Rtg2p is a protein involved in many cellular processes related to aging, and our structural map may provide the means to analyze more Rtg2p functions. One of these functions is the participation on the complex SLIK, a specialized form of the coactivator complex SAGA [15]. It was proposed that Rtg2p expands SAGA repertoire of gene modulation that includes several genes involved in stress response [53]. Our mutants could be used to probe the SAGA-Rtg2p association and their participation in chromatin remodeling. Likewise, the contribution of Rtg2p to the expansion/contraction of CTG•GAC triplets [14] may be studied by mutants designed by DRCN, and that may shed light to the mechanism shared in neurodegenerative diseases like Huntington´s disease. Another important trait of genomic instability modulated by Rtg2p activity is the formation of the extrachromosomal ribosomal-DNA (ERC) circles, that accumulate in cells with aging [16]. In yeast *rtg2*Δ background ERCs accumulate and eventually kill cells [17]. Because Rtg2p function that suppresses ERCs formation is observed only when the protein is not involved in propagating retrograde signaling [16], further studies of the mutants described here could be very informative, especially those that do not activate retrograde signaling but still extends longevity. The usefulness of the mutants could also be extended to the role of Rtg2p in the acidification of the vacuole in cells deficient in cardiolipin [54]. Elevation of vacuole pH is also strongly related to aging and mitochondrial dysfunction [55]. Overall, these works indicate that Rtg2p is very important for the longevity of *S*. *cerevisiae*, and its functional crosstalks with several other pathways of the cell could also be structurally mapped in Rtg2p by a similar approach shown here.

## Supporting information

S1 Fig Structural superposition of Rtg2p and the exopolyphosphatase of *Agrobacterium fabrum* strain C58. Structural superposition of Rtg2p Robetta model (red) and the crystal structure of the exopolyphosphatase of *Agrobacterium fabrum* (orange; cover structural similarity 67.6%; PDB ID 3HI0). Rtg2p unaligned regions are shown in blue. Superposition was performed with TopMatch (https://topmatch.services.came.sbg.ac.at/).
(TIF)

S2 Fig Location of conserved a residues in Rtg2p N-terminal structure. Conserved residues with conservation highest than 90% in PF02541 family were obtained by DRCN analysis. These residues were modified by site-directed mutagenesis, and are indicated in sticks representation in Rtg2p N-terminal structural model.
(TIF)

S3 Fig Lack of mitochondrial DNA does not extends longevity in PSY142 background. Fifty cells of each strain were aligned on YPD and daughter cells removed from mothers to construct survival curves from at least two independent experiments. Mean and maximum longevity are indicated between parentheses (mean, maximum). Statistical analyses were performed by Mann-Whitney test; p <0.895 [rho0] vs. [rho+].
(TIF)

S4 Fig *RTG2* mutants show normal growth on rich YPD medium, except for a mild effect in *RTG2-E106H*. Growth performance of strains on rich liquid medium YPD. Wild type, *rtg2*Δ, and *RTG2* mutant strains were grown on YPD until saturation, and diluted to A_600_ = 0.1 in 200 μL of rich medium. The cells were incubated at 30°C, 160 rpm, for 24 h and growth monitored in a INFINITY PRO 200 (Tecan) microplate reader.
(TIF)

S5 Fig *RTG2* point mutations that failed to increase the RLS when compared to PSY142 ρ^0^. **(A)** *RTG2* ρ^0^ point mutants show *CIT2* expression comparable to that of *rtg2*Δ ρ^0^ strain. Petite strains, generated from wild type, *rtg2*Δ and *RTG2* point mutants, were grown on YPR medium until to analyze *CIT2-LacZ* expression. β-galactosidase assays were performed in triplicate as described in the Materials and Methods section. *RTG2* mutant ρ^0^ strains *RTG2-L56G* ρ^0^ **(B)**, mutants in surface residues *RTG2-E106H* ρ^0^ and *RTG2-E106A* ρ^0^ **(C)**, and mutants in putative residues involved in Mg^2+^ ion binding, *RTG2-D158A* ρ^0^ **(D)** and *RTG2-S163E* ρ^0^ **(E)** failed to increase RLS, when compared to WT ρ^0^. In RLS assays, fifty cells of each strain were aligned on YPR and daughter cells were removed from mothers to construct survival curves from at least two independent experiments. The mean and maximum longevity are indicated between parentheses (mean, maximum). Statistical significance between samples is summarized in Table 3.
(TIF)

S1 Table Oligonucleotides used to construct *RTG2* mutants and to confirm integration locus. (DOCX)

S2 Table Validation by Ramachandran diagram of three-dimensional models of Rtg2p obtained in five different servers. (DOCX)

S3 Table Validation by Verify3D of three-dimensional models of Rtg2p obtained in five different servers. ^1^Percentage of the residues with an averaged 3D-1D score ≥ 0.2.
(DOCX)

S4 Table Residues with conservation highest than 90% in PF02541 family. In red are positions that diverge from the conservation observed in the majority of the sequences f the alignment.
(DOCX)
