# Supplementary figures and images for: Structural and functional mapping of Rtg2p determinants involved in retrograde signaling and aging of Saccharomyces cerevisiae
Source: PLoS One. 2017 May 4;12(5):e0177090. doi: 10.1371/journal.pone.0177090 (PMC5417653; doi:10.1371/journal.pone.0177090)

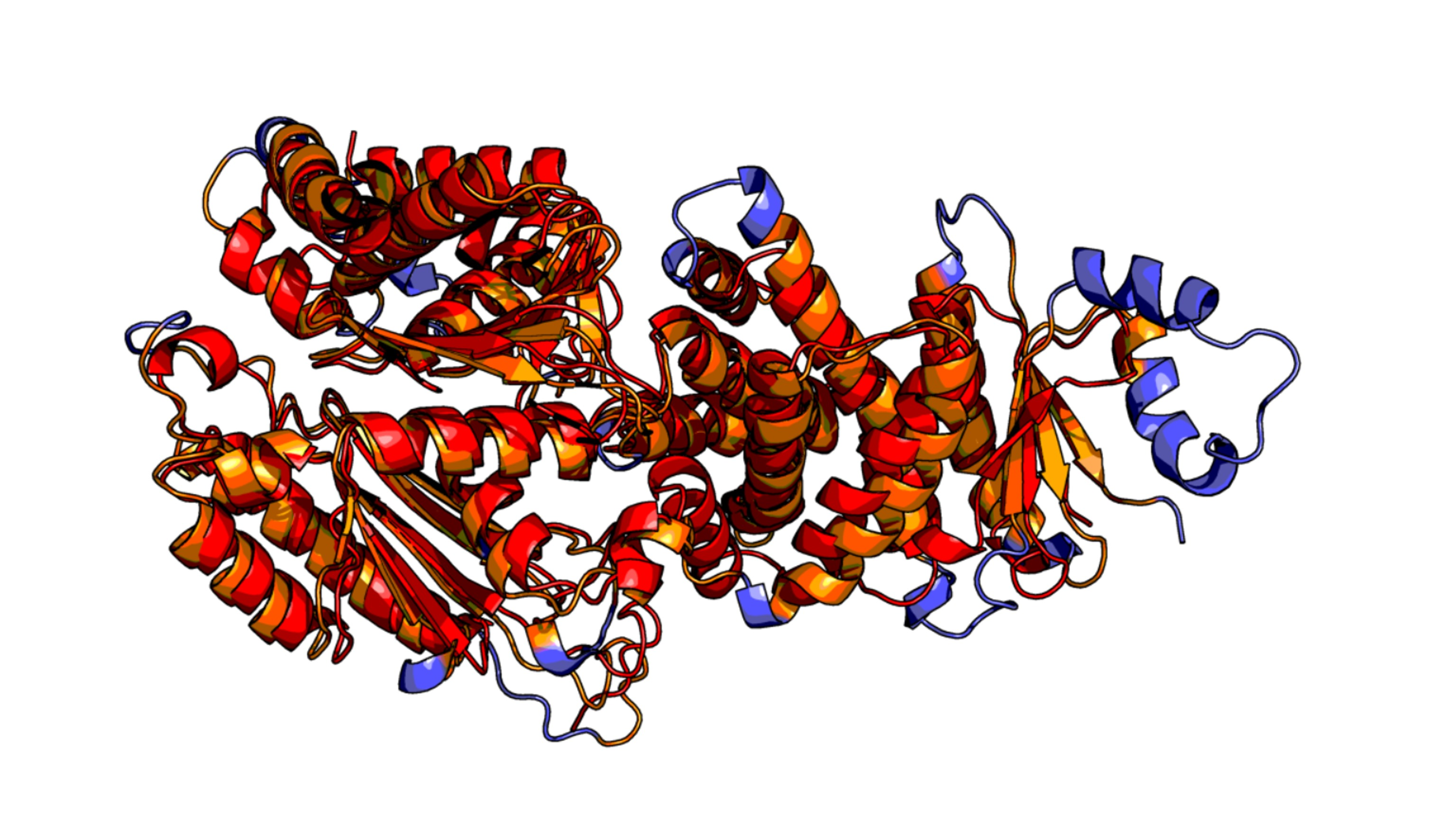

Supplement: S1 Fig — Structural superposition of Rtg2p Robetta model (red) and the crystal structure of the exopolyphosphatase of Agrobacterium fabrum (orange; cover structural similarity 67.6%; PDB ID 3HI0). Rtg2p unaligned regions are shown in blue. Superposition was performed with TopMatch (https://topmatch.services.came.sbg.ac.at/). (TIF) [file pone.0177090.s001.tif]

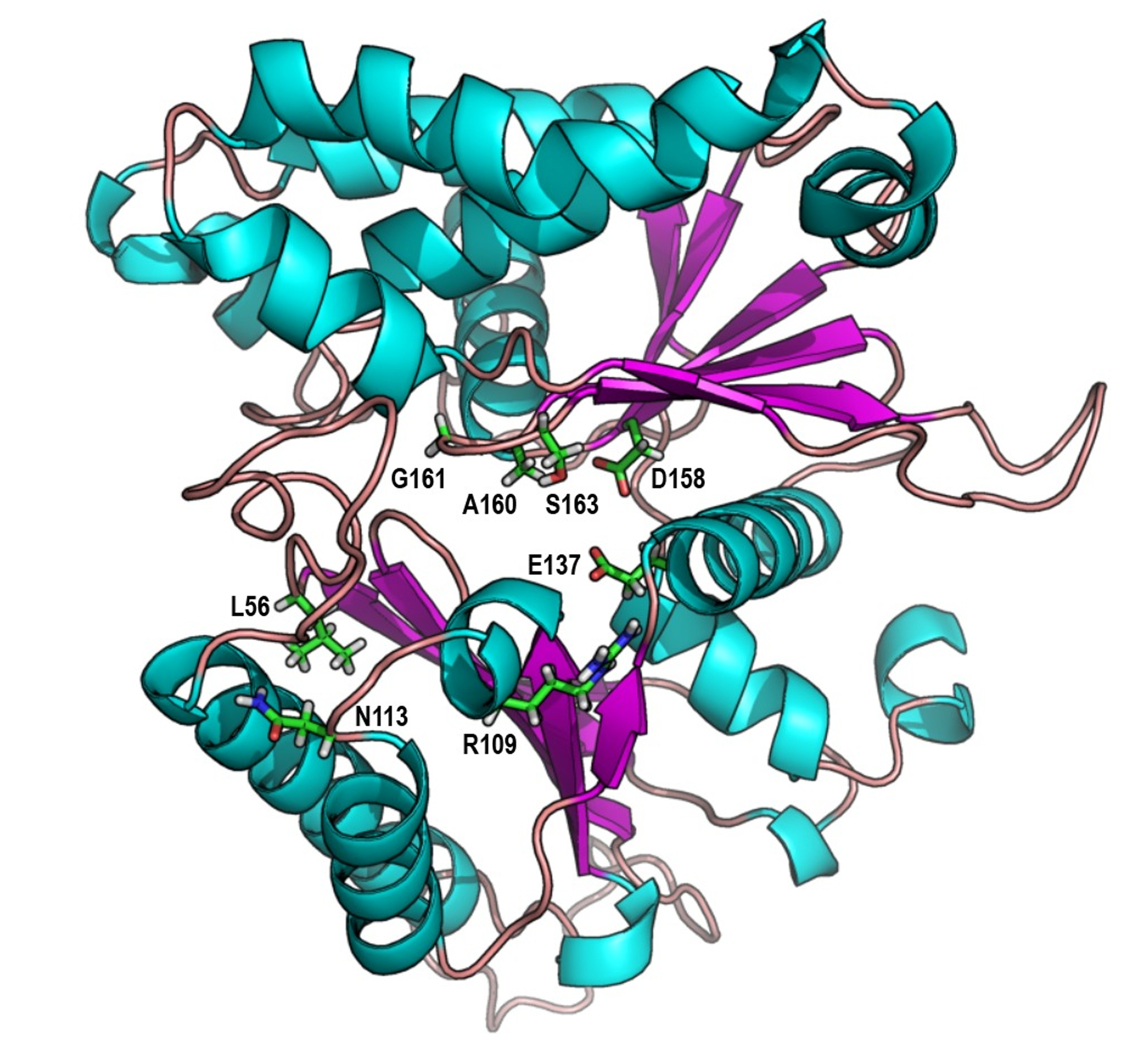

Supplement: S2 Fig — Conserved residues with conservation highest than 90% in PF02541 family were obtained by DRCN analysis. These residues were modified by site-directed mutagenesis, and are indicated in sticks representation in Rtg2p N-terminal structural model. (TIF) [file pone.0177090.s002.tif]

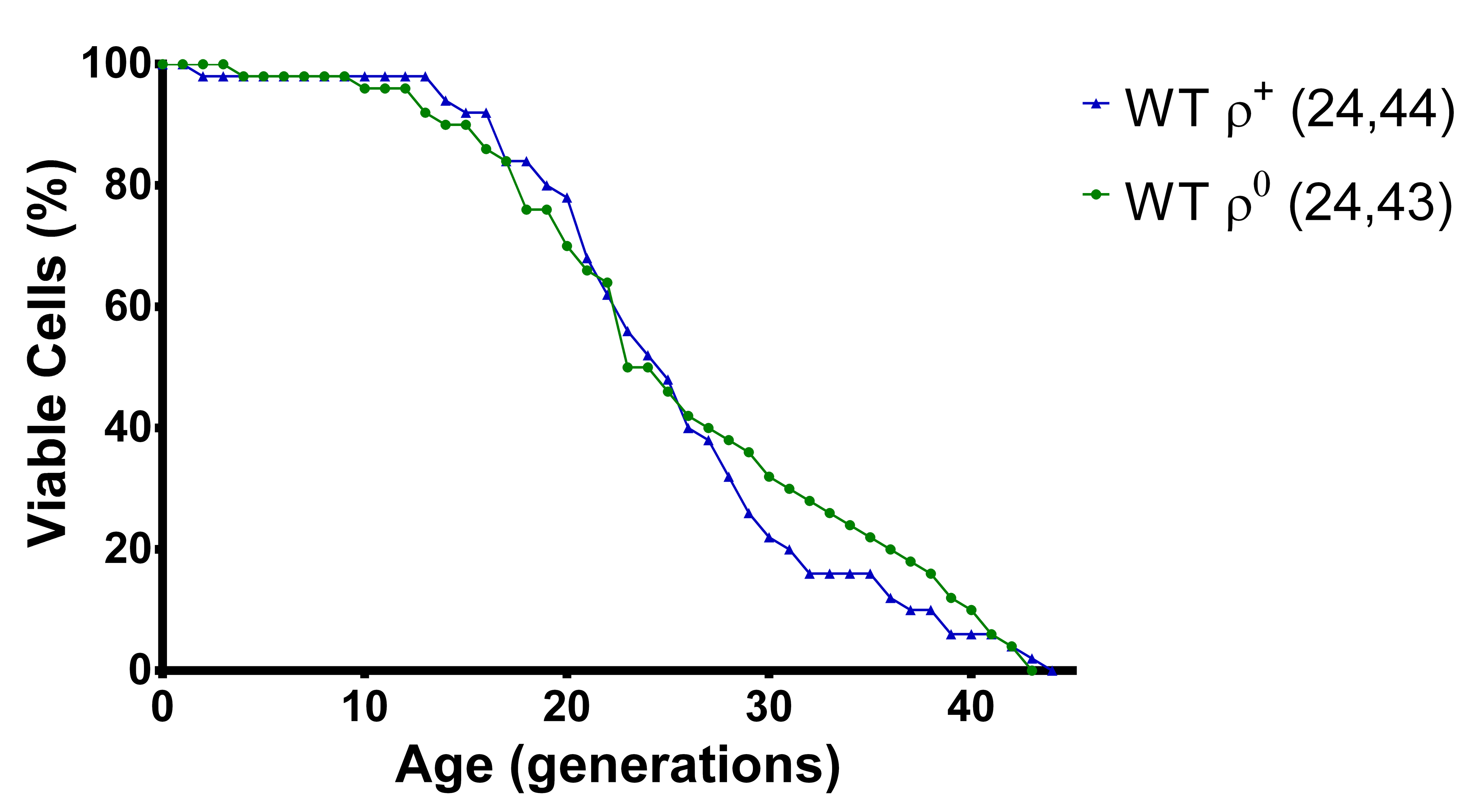

Supplement: S3 Fig — Fifty cells of each strain were aligned on YPD and daughter cells removed from mothers to construct survival curves from at least two independent experiments. Mean and maximum longevity are indicated between parentheses (mean, maximum). Statistical analyses were performed by Mann-Whitney test; p <0.895 [rho0] vs. [rho+]. (TIF) [file pone.0177090.s003.tif]

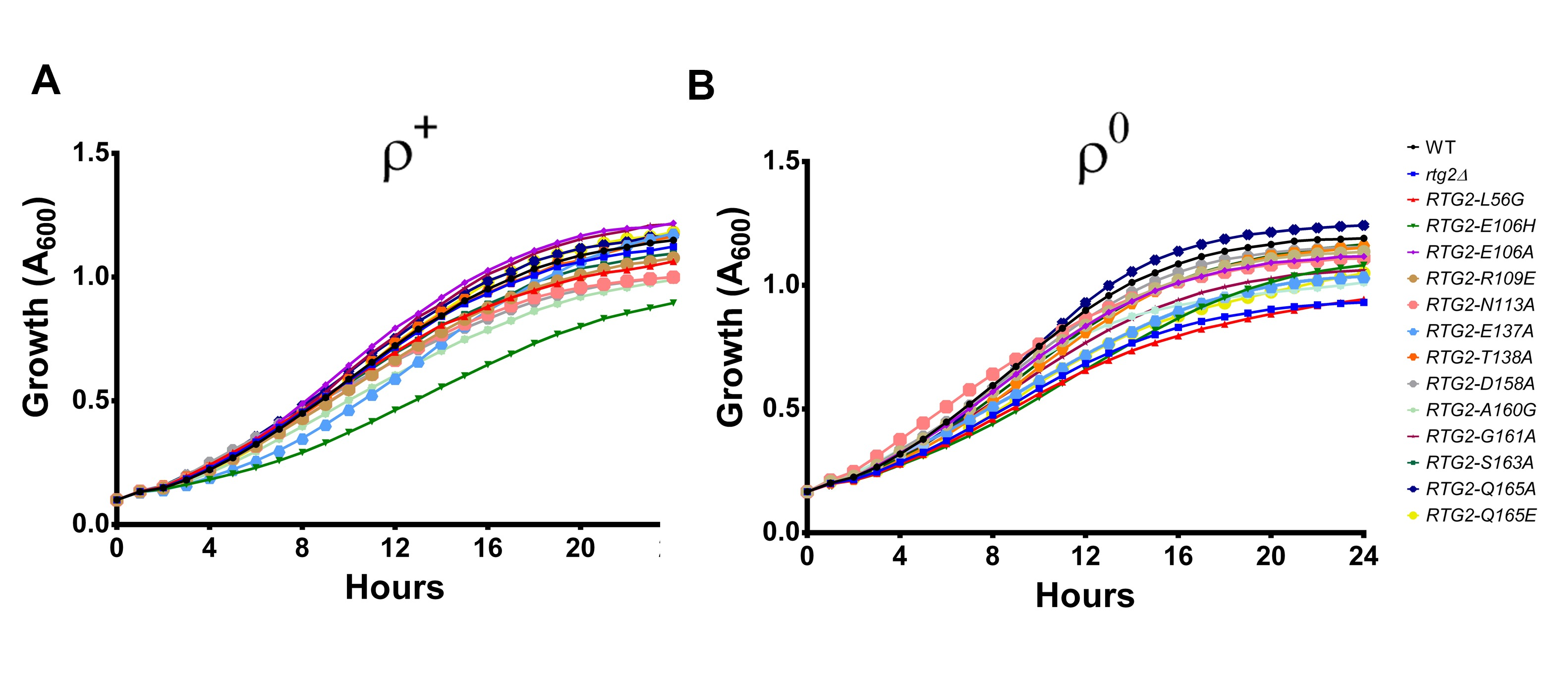

Supplement: S4 Fig — Growth performance of strains on rich liquid medium YPD. Wild type, rtg2Δ, and RTG2 mutant strains were grown on YPD until saturation, and diluted to A600 = 0.1 in 200 μL of rich medium. The cells were incubated at 30°C, 160 rpm, for 24 h and growth monitored in a INFINITY PRO 200 (Tecan) microplate reader. (TIF) [file pone.0177090.s004.tif]

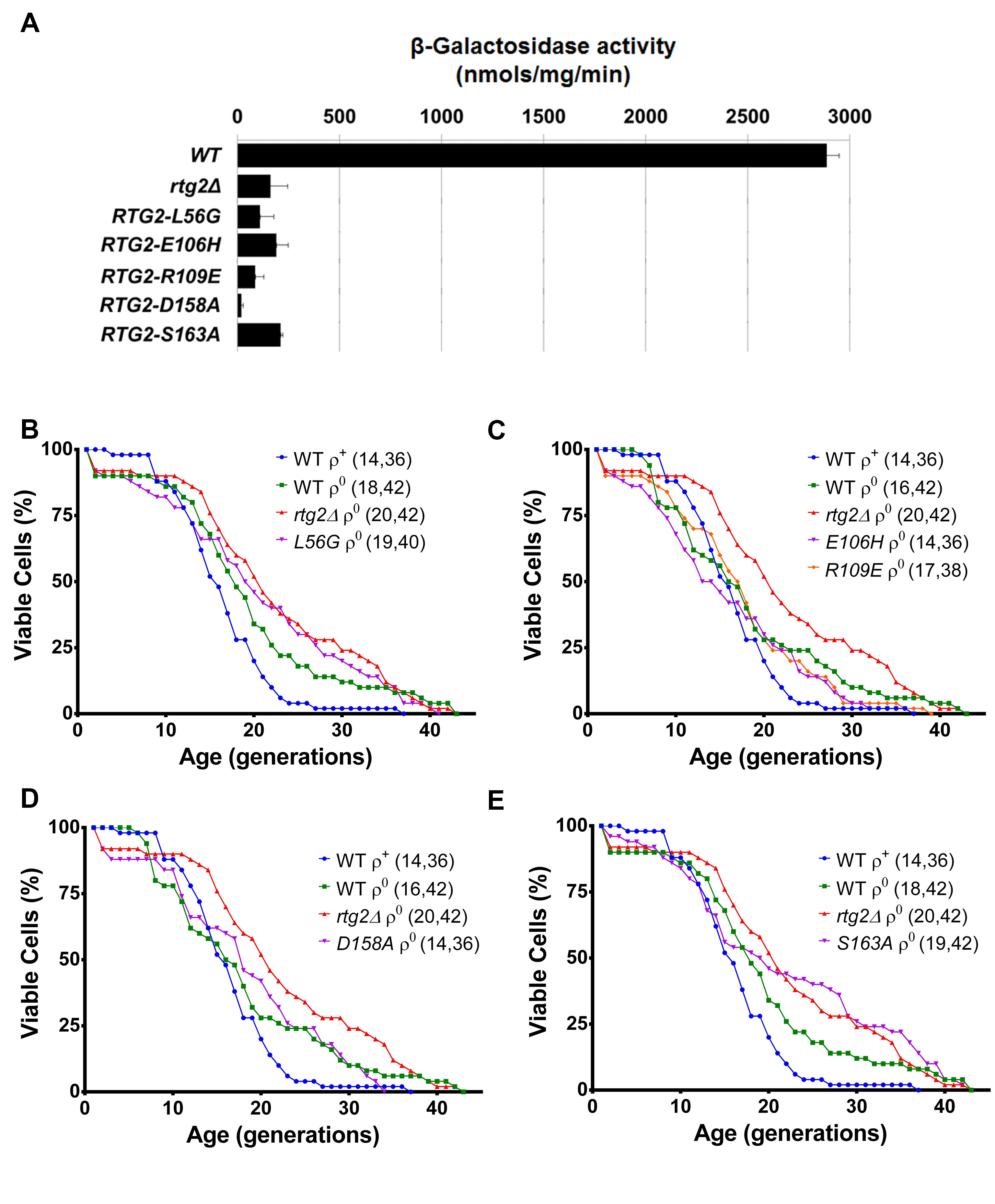

Supplement: S5 Fig — (A) RTG2 ρ0 point mutants show CIT2 expression comparable to that of rtg2Δ ρ0 strain. Petite strains, generated from wild type, rtg2Δ and RTG2 point mutants, were grown on YPR medium until to analyze CIT2-LacZ expression. β-galactosidase assays were performed in triplicate as described in the Materials and Methods section. RTG2 mutant ρ0 strains RTG2-L56G ρ0 (B), mutants in surface residues RTG2-E106H ρ0 and RTG2-E106A ρ0 (C), and mutants in putative residues involved in Mg2+ ion binding, RTG2-D158A ρ0 (D) and RTG2-S163E ρ0 (E) failed to increase RLS, when compared to WT ρ0. In RLS assays, fifty cells of each strain were aligned on YPR and daughter cells were removed from mothers to construct survival curves from at least two independent experiments. The mean and maximum longevity are indicated between parentheses (mean, maximum). Statistical significance between samples is summarized in Table 3. (TIF) [file pone.0177090.s005.tif]
