# Supplementary material for: Structural and functional mapping of Rtg2p determinants involved in retrograde signaling and aging of Saccharomyces cerevisiae
Source: PLoS One. 2017 May 4;12(5):e0177090. doi: 10.1371/journal.pone.0177090 (PMC5417653; doi:10.1371/journal.pone.0177090)
Supplement: S1 Table — (DOCX) [file pone.0177090.s006.docx]

**S1 Table. Oligonucleotides used to construct *RTG2* mutants and to confirm integration locus.**

| Oligonucleotide identification | Sequence (5′→3′) | Ta (°C) |
| --- | --- | --- |
| Rtg2-597 | GCG GGT GAA CAA GTC CTA AA | 55 |
| Rtg2-2217 | TCA CTA GAC GAC TAC CCA ATC A | 55 |
| rtg2-URF13-KanMX-F | CTT TAC TAA GGA TTG TTT TGA ACG AAA AGT GTA GGC GTG CCA CAA AAC AGC TAT GAC CAT GAT TAC | 55 |
| rtg2-URF13-KanMX-R | AAG GAT TTC GTA TTT ATT GTT CAA GTA TTT AAA GAC TAG ATG TCT AAA ACG ACG GCC AGT GAA TTC | 55 |
| KanB | CTG CAG CGA GGA GCC GTA AT | 56 |
| KanC | TGA TTT TGA TGA CGA GCG TAA T | 49 |
| Rtg2-F | TCA GCT AAG CTT GAA AGG AAG AAA TCA TCA AAG | 56 |
| Rtg2-R | CTG ACT GGA TCC CTA CTT ATG TGA ACA TG | 56 |
| L56G-F | GTT GGT CTT TCT GGA TAC GAA GTT CAA | 55 |
| L56G-R | TTG AAC TTC GTA TCC AGA AAG ACC AAC | 55 |
| E106H-F | GTA ATT GCA ACA CAT GCC ACG CGA GAT | 55 |
| E106H-R | ATC TCG CGT GGC ATG TGT TGC AAT TAC | 55 |
| E106A-F | GTA ATT GCA ACA GCA GCC ACG CGA GAT | 55 |
| E106A-R | ATC TCG CGT GGC TGC TGT TGC AAT TAC | 55 |
| R109E-F | ACA GAA GCC ACG GAA GAT GCT ATT AAC | 55 |
| R109E-R | GTT AAT AGC ATC TTC CGT GGC TTC TGT | 55 |
| N113A-F | CGA GAT GCT ATT GCC GCG GAT GAA TTT G | 55 |
| N113A-R | CAA ATT CAT CCG CGG CAA TAG CAT CTC G | 55 |
| E137A-F | GGC CAG GAA GAT GCA ACT AGG GTC GGC | 55 |
| E137A-R | GCC GAC CCT AGT TGC ATC TTC CTG GCC | 55 |
| T138A-F | GGT GGT AGT ACT GAG TTA TCA TGG GTA | 55 |
| T138A-R | GGT GGT AGT ACT GAG TTA TCA TGG GTA | 55 |
| D158A-F | GGT CTA TAT CTA GCT GTG GCA GGT GGT | 55 |
| D158A-R | ACC ACC TGC CAC AGC TAG ATA TAG ACC | 55 |
| A160G-F | TAT CTA GAT GTG GGA GGT GGT AGT ACT | 55 |
| A160G-R | AGT ACT ACC ACC TCC CAC ATC TAG ATA | 55 |
| G161A-F | CTA GAT GTG GCA GCT GGT AGT ACT CAG | 55 |
| G161A-R | CTG AGT ACT ACC AGC TGC CAC ATC TAG | 55 |
| S163A-F | GTG GCA GGT GGT GCT ACT CAG TTA TCA | 55 |
| S163A-R | TGA TAA CTG AGT AGC ACC ACC TGC CAC | 55 |
| Q165E-F | GGT GGT AGT ACT GAG TTA TCA TGG GTA | 55 |
| Q165E-R | TAC CCA TGA TAA CTC AGT ACT ACC ACC | 55 |
| Q165A-F | GGT GGT AGT ACT GCG TTA TCA TGG GTA | 55 |
| Q165A-R | TAC CCA TGA TAA CGC AGT ACT ACC ACC | 55 |
