# Supplementary material for: Structural and functional mapping of Rtg2p determinants involved in retrograde signaling and aging of Saccharomyces cerevisiae
Source: PLoS One. 2017 May 4;12(5):e0177090. doi: 10.1371/journal.pone.0177090 (PMC5417653; doi:10.1371/journal.pone.0177090)
Supplement: S2 Table — (DOCX) [file pone.0177090.s007.docx]

**S2 Table. Validation by Ramachandran diagram of three-dimensional models of Rtg2p obtained in five different servers.**

| **Server** | **Ramachandram diagram** | | |
| --- | --- | --- | --- |
|  | **Favoured regions** | **Additional allowed regions** | **Generously allowed regions** |
| **Robetta** | 90,2% | 6,9% | 1,5% |
| **Swiss-Model** | 86,2% | 11,4% | 1,2% |
| **Phyre 2** | 86,3% | 12,0% | 0,7% |
| **EsyPred3D** | 84,7% | 11,8% | 2,6% |
| **I-Tasser** | 75,9% | 17,2% | 6,8% |
