# Supplementary material for: Structural and functional mapping of Rtg2p determinants involved in retrograde signaling and aging of Saccharomyces cerevisiae
Source: PLoS One. 2017 May 4;12(5):e0177090. doi: 10.1371/journal.pone.0177090 (PMC5417653; doi:10.1371/journal.pone.0177090)
Supplement: S3 Table — 1Percentage of the residues with an averaged 3D-1D score ≥ 0.2. (DOCX) [file pone.0177090.s008.docx]

**S3 Table. Validation by Verify3D of three-dimensional models of Rtg2p obtained in five different servers.**

| Server | Structural compatibility^1^ |
| --- | --- |
| Robetta | 82,48% |
| Swiss-Model | 74,69% |
| Phyre 2 | 76,34% |
| I-Tasser | 71,09% |
| EsyPred3D | 56,27% |

^1^Percentage of the residues with an averaged 3D-1D score ≥ 0.2.
