# Supplementary material for: Structural and functional mapping of Rtg2p determinants involved in retrograde signaling and aging of Saccharomyces cerevisiae
Source: PLoS One. 2017 May 4;12(5):e0177090. doi: 10.1371/journal.pone.0177090 (PMC5417653; doi:10.1371/journal.pone.0177090)
Supplement: S4 Table — In red are positions that diverge from the conservation observed in the majority of the sequences f the alignment. (DOCX) [file pone.0177090.s009.docx]

**Table S4. Residues with conservation highest than 90% in PF02541 family.**

|  |  |  |  |  | Conservation of residues in the family  (%) | | | | |  |  |  |  |  |
| --- | --- | --- | --- | --- | --- | --- | --- | --- | --- | --- | --- | --- | --- | --- |
| UNIPROT  code | **Organism** | L110 (92.8) | T204 (100.0) | R209 (99.5) | A215 (93.3) | N217 (92.3) | E262 (100.0) | D411 (99.3) | G418 (99.8) | G419 (99.7) | S421 (97.9) | G495 (100.0) | R863 (99.7) | G936 (98.6) |
| RTG2_YEAST | Saccharomyces cerevisiae | L56 | T105 | R109 | A111 | N113 | E137 | D158 | **A160** | G161 | S163 | G188 | R303 | G337 |
| A9CJF9_AGRT5 | *Agrobacterium fabrum* | L50 | T92 | R96 | A98 | N100 | E124 | D145 | G147 | G148 | S150 | G170 | R268 | G301 |
| O67040_AQUAE | Aquifex aeolicus | L45 | T87 | R91 | A93 | N95 | E119 | D141 | G143 | G144 | S146 | G166 | R267 | G300 |
| PPX_ECO57 | Escherichia coli O157 | L47 | T89 | R93 | A95 | N97 | E121 | D143 | G145 | G146 | S148 | G168 | R267 | G300 |
| PPX_ECOLI | Escherichia coli K12 | L47 | T89 | R93 | A95 | N97 | E121 | D143 | G145 | G146 | S148 | G168 | R267 | G300 |
| Q11YA9_CYTH3 | Cytophaga hutchinsonii | L39 | T81 | R85 | **G87** | N89 | E113 | D136 | G138 | G139 | S141 | G161 | R268 | G301 |
| Q8G5J2_BIFLO | Bifidobacterium longum | L43 | T85 | R89 | A91 | N93 | E117 | D142 | G144 | G145 | S147 | G173 | R278 | G317 |

In red are positions that diverge from the conservation observed in the majority of the sequences f the alignment.
